# A Comparative and Comprehensive Review of Antibody Applications in the Treatment of Lung Disease

**DOI:** 10.3390/life12010130

**Published:** 2022-01-17

**Authors:** Yuefeng Wu, Hai Song

**Affiliations:** 1 Zhejiang University-University of Edinburgh Institute, Zhejiang University School of Medicine, Zhejiang University, Hangzhou 314400, China; 2 Life Sciences Institute, Zhejiang University, Hangzhou 310000, China; haisong@zju.edu.cn

**Keywords:** antibody, COVID-19, cancer, immunotherapy, bioinformatics

## Abstract

Antibodies are a type of protein produced by active B cells in response to antigen stimulation. A series of monoclonal antibodies and neutralizing antibodies have been invented and put into clinical use because of their high therapeutic effect and bright developing insight. Patients with cancer, infectious diseases, and autoimmune diseases can all benefit from antibody therapy. However, the targeting aspects and potential mechanisms for treating these diseases differ. In the treatment of patients with infectious diseases such as COVID-19, neutralizing antibodies have been proposed as reliable vaccines against COVID-19, which target the ACE2 protein by preventing virus entry into somatic cells. Monoclonal antibodies can target immune checkpoints (e.g., PD-L1 and CTLA-4), tyrosine kinase and subsequent signaling pathways (e.g., VEGF), and cytokines in cancer patients (e.g. IL-6 and IL-1β). It is debatable whether there is any connection between the use of antibodies in these diseases. It would be fantastic to discover the related points and explain the burden for the limitation of cross-use of these techniques. In this review, we provided a comprehensive overview of the use of antibodies in the treatment of infectious disease and cancer patients. There are also discussions of their mechanisms and history. In addition, we discussed our future outlook on the use of antibodies.

## 1. Introduction

**Figure 1:**
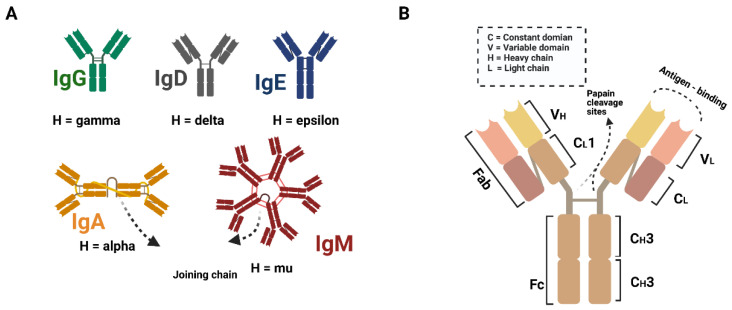
Pattern diagram of antibody subtypes and structure. (**A**) IgG is the most abundant type of antibody. IgM takes 5–10% of total antibodies in the serum. IgA accounts for 10–15% of total immunoglobulins in the serum. IgD (0.2%) and IgE (0.3%) only account for a minor portion of whole serum antibodies. (**B**) The structure of an immunoglobulin.

By producing immunoglobulins against foreign antigens, B cells play a critical role in the adaptive immune response. Immunoglobins, also known as antibodies, are a type of glycosylated protein molecule that appears on the surface of B cells and is secreted into the body fluid to perform a neutralizing function by binding to specific antigens. Immunoglobulins are classified into five subtypes: IgM, IgD, IgG, IgA, and IgE, respectively (Figure 1A). These subtypes are classified based on the structure of the immunoglobulin (Figure 1B). The immunoglobulin is made up of four proteins that are linked together by disulfide bonds. Based on molecule weight, these four proteins are referred to as "heavy chain" and "light chain". The antigen-binding side is also formed by the combined efforts of the heavy and light chains at the *N*-terminus. Furthermore, the classification is based on the *C*-terminus regions of the heavy chains. Although the *C*-terminus is not involved in antigen binding, the effector functions are essential [1].

Antibodies perform their duty in the following ways: (1) Neutralizing the corresponding targets. (2) Activating immune cells by binding to Fc receptors. (3) Activating or deactivating classical pathways. Under normal circumstances, B cells collaborate with other immune system components. There are, of course, exceptions. Autoimmune disease occurs when the immune system becomes overly active. In patients with systemic lupus erythematosus, for example, IgG levels are significantly higher than normal, which may be caused by polysaccharide antigens or cytokines (e.g. IL-4 and IL-21). Interestingly and importantly, the autoimmune disease may be caused by microbial infection. Jasemi S, et al. showed the impact of infection by microbial to rheumatoid arthritis etiopathogenesis. They measured the titers of antibodies derived from Porphyromonas gingivalis, Aggregatibacter actinomycetemcomitans, Mycobacterium avium subsp. Paratuberculosis, and Epstein–Barr virus were compared with RA descriptors. Their study demonstrates the significance of increased humoral response in RA pathogenesis, which gives clues for RA antibody therapy [2]. Meanwhile, Bo M, et al. successfully employed bioinformatics analysis [3] and a mouse model [4] to identify and validate the potential biomarker and therapeutic point for the autoimmune disease. They reported IRF5 as a potential target of autoimmune response triggered by microbial infection, indicating cytokines as an essential direction for antibody-based drug development.

Cancer patients may have had a weakened immune system, which should have eliminated the cancerous cells. There is already a wide range of antibody products available for cancer treatment. All three of the mechanisms mentioned above have been used, and they will be introduced and discussed further below.

In air-breathing vertebrates, the lung is an essential organ for gas exchange. The lung is located in the chest cavity and is protected by a thin membrane called the pleura. The main bronchus connects the left and right lungs to the trachea. The heart is also linked to the lungs via pulmonary arteries and veins, which allow blood to flow in and out of the lungs. The actual gas exchange occurs in the alveoli, which are located at the end of the subdividing bronchus [5]. In addition to gas exchange, water, and alcohol excretion, the lung undergoes a wide range of chemical synthesis and degradation.

The lung, on the other hand, is prone to infection and injury, including pneumoconiosis, systemic lupus erythematosus (SLE), non-small cell lung cancer, and small cell lung cancer. Fortunately, antibodies can be used effectively to treat the aforementioned diseases.

It has been roughly half a century since the establishment of the of B cells [6] and several decades since the discovery of therapeutic antibodies. It is critical to provide a comprehensive review of the progress made in the application of antibodies in the treatment of infectious disease and cancer in this section. We will discuss COVID-19, asthma, and lung cancers in this article. Additionally, while the application of antibodies is still limited today, the convergence of the mechanisms underlying these three types of disease is significant, as it will provide insight for future antibody invention.

## 2. The History of Antibodies Application in Clinical Therapy

Several different terminologies in antibody classification have been developed in order to broaden the scientific discussion and improve interpretation. These terminologies are based on their function, origin, production, and so on. The antibodies that have the ability to “neutralize” virus infection are referred to as “neutralizing antibodies.” Monoclonal antibodies are antibodies produced by a single-origin B cell with hybridoma. The antibodies whose genetics have been altered by humans are referred to as genetically engineered antibodies. Finally, antibody–drug conjugation refers to antibody–drug interactions. In the sections that follow, we will use different terminology to discuss their histories and mechanisms.

### 2.1. The General Mechanism of Neutralizing Antibody Fighting against Virus 

**Figure 2:**
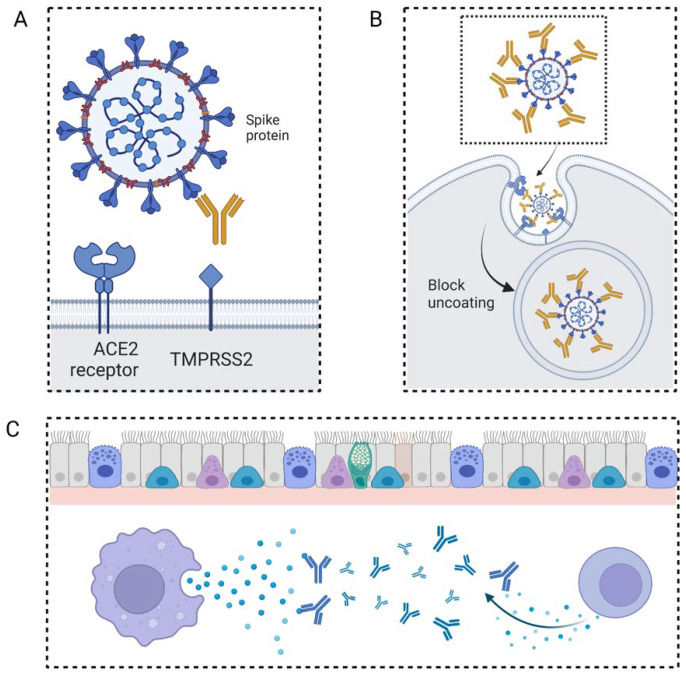
Pattern diagram of neutralizing antibody mechanism. (**A**) Preventing the combination of pathogens and somatic cell surface receptors. (**B**) Preventing virus uncoating in somatic cells. (**C**) Influencing the cytokines in the serum.

As part of an immune response, neutralizing antibodies bind to specific corresponding antigens. Furthermore, unlike other antibodies, they react with viruses or other pathogens, as well as some cytokines. The neutralizing antibodies, on the other hand, are ineffective against extracellular bacteria because antibody binding does not prevent bacteria from multiplying (Figure 2). Finally, they can disable pathogens or somatic cell functions, thereby preventing human infections and illnesses [7]. Infection prevention is achieved by the following means: (1) Avoiding the combination of pathogens and somatic cell surface receptors, e.g., monoclonal blocking antibody neuropilin-1, which is known for its ability to bind furin-cleaved substrate. Blocking these receptors or entry cofactors may reduce the pathogens’ infectivity [8]. (2) Preventing virus uncoating in somatic cells. Uncoating or capsid core disassembly is essential before the viral genomic DNA integrates into the host chromosomes. The structure of some viral coats was previously revealed [9]—even the cellular location of uncoating was identified for some viruses as well [10]. Zhang et al. proposed an essential method for treating enterovirus 71 infection by preventing the uncoating process and stopping the hole formation on the membrane [11]. (3) By influencing the cytokines in the serum. Since the cytokines play a fundamental role in the immune response regulation, the abnormal phenotype may be adjusted into the normal range through cytokine mimicry or neutralization.

### 2.2. The History of Neutralizing Antibodies

**Figure 3:**
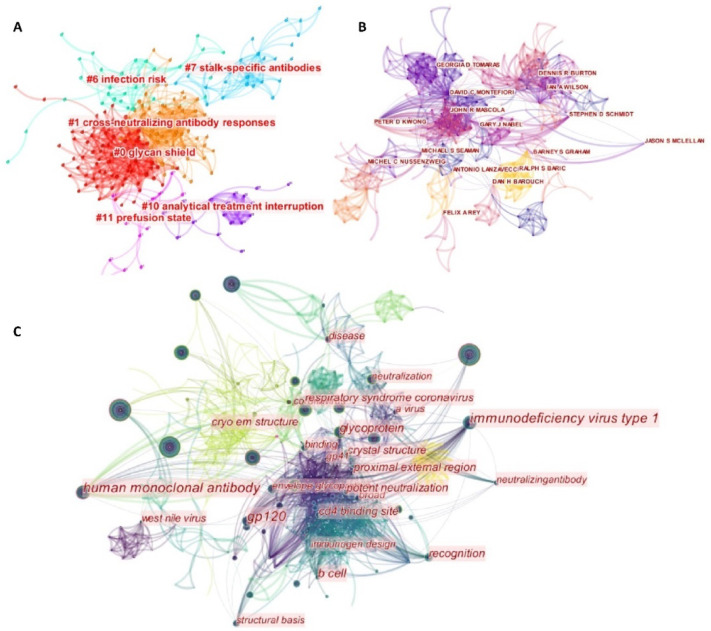
Clustering analysis of Web of Science abstracts (2010–present) on the subject of neutralizing antibodies. (**A**) The auto-clusters are labeled with an abstract index. (**B**) The auto clusters are labeled with the author index. (**C**) Clustering of keywords among neutralizing antibody-related papers.

In fact, it has been a long time since the first clinical application of neutralizing antibodies. Emil Adolf von Behring provided an effective serum treatment for patients with diphtheria in 1891. Soon afterward, it was applied to treat diphtheria. However, due to the low titer and heterozygous of effective antibodies in the serum, the application of this “old school” technique was limited at that time. During the 1970s, with the development of effective antibody identification (flow cytometry) and purification, it had become easy to generate sufficient effective antibodies from the mouse spleen. Meanwhile, optimization strategies, such as Fc modification, were applied. The application of neutralizing antibodies had seen a significant victory in the fight against the Zaire ebolavirus outbreak. Nowadays, with the development of computational biology and the discovery of immunology, more advances have been made, and many different monoclonal antibodies have been invented [12]. The keyword ‘neutralizing antibody’ was searched in the Web of Science to obtain an overview of the history of the development of neutralizing antibodies. Only papers tagged with the “article” were retrieved for analysis. The time span of available literature was enormous (1917–2021). All Web of Science core papers published from 2010 until now were visualized with CiteSpace [13]. Based on a cluster analysis of terms from the abstracts (Figure 3A), it was found that the terms infection risk, stalk-specific antibodies, cross-neutralizing antibody responses, glycan shield, analytical treatment interruption, and prefusion state have repeatedly appeared, indicating that these fields are research hotpots. Figure 3B shows the connection between authors who have published papers related to neutralizing antibodies, providing an insight into the present review. Figure 3C depicts the relationships between the various keywords. It not only indicates previous fields of research but also provides a unique perspective on future research directions, including the structural basis, human monoclonal antibodies, B cells, the CD4 binding site, envelop glycoproteins, potent neutralization, the cryo-electron microscopy (Cryo-EM) structure, and the proximal external region. For future research, the terms described above can be separated into three categories. First, "cross-neutralizing antibody reactions" should be considered while using newly developed antibody medicines. The following section in his review will give an example of how to cross-use SARS-CoV antibody for SARS-CoV-2 treatment. From a scientific standpoint, technological advancements in structural understanding (Cryo-EM) aided in the study of infection mechanisms and the development of appropriate medications. Meanwhile, more research on the binding ability measurement and recognition mechanism may be advantageous in terms of theoretical development.

### 2.3. The Detailed Mechanism of Monoclonal Antibody Cytotoxicity

Kohler and Milstein described a technology in 1975 that can continuously produce a predefined antibody with high specificity by fusing mouse myeloma with mouse spleen cells. Because these antibodies were derived from mice, they were difficult to use in the clinic. Later, humanized monoclonal antibodies were created by combining human Fc domains with mouse variable regions.

**Figure 4:**
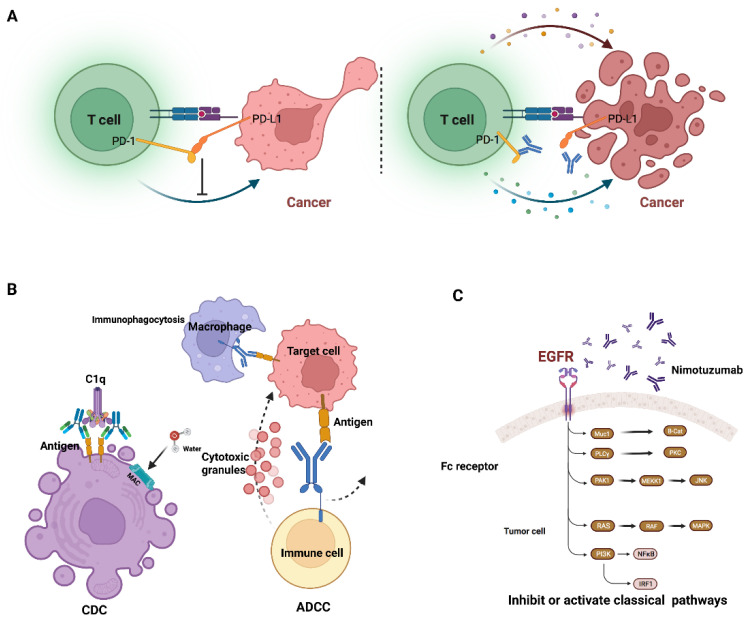
Pattern diagram of monoclonal antibody mechanism. (**A**) Immune checkpoint (PD-1/PD-L1) mechanism (**B**) antibody-dependent cellular cytotoxicity (ADCC), and complement-dependent cytotoxicity (CDC) (**C**) affect the signaling pathway within tumor cells (created with Biorender).

Some clinically used mAbs interact with the immune system via antibody-dependent cellular cytotoxicity (ADCC) [14] and complement-dependent cytotoxicity (CDC) [15]. ADCC is a mechanism in which antibodies bind to specific types of cells, directing effector cells to kill these antibody-coated cells. CDC is a mechanism by which the complex formed by the complement protein C1q and the Fc domain of mAbs, also known as the membrane attack complex (MAC), forms pores on cell membranes [16] (Figure 4B).

Some clinically used mAbs bind to cell surface antigens and affect the signaling pathway within tumor cells. Subsequently, it was discovered that some membrane receptors, particularly growth factor receptors, have an unusually high number in tumor cells. mAbs targeting these receptors were identified as an effective therapy for normalizing cell division and differentiation (Figure 4C).

Some clinically used mAbs work by modulating the immune system. Antibodies that target PD-1/PD-L1 and CTLA-4 have already shown significant efficacy in treating patients with a variety of cancers (Figure 4A). The immune checkpoint plays a preventative role in immune response, which initially aids in the survival of healthy cells in the body. Immune checkpoint proteins link the targeted cell to the immune cell and send the terminal signal to the immune cell. These mAbs that target immune checkpoints can prevent the transmission of these terminal signals. Downregulation of the immune system is beneficial in the treatment of autoimmune disease, whereas upregulation is beneficial in the treatment of cancer patients.

### 2.4. The Mechanism of Antibody–Drug Conjugates (ADC)

**Figure 5:**
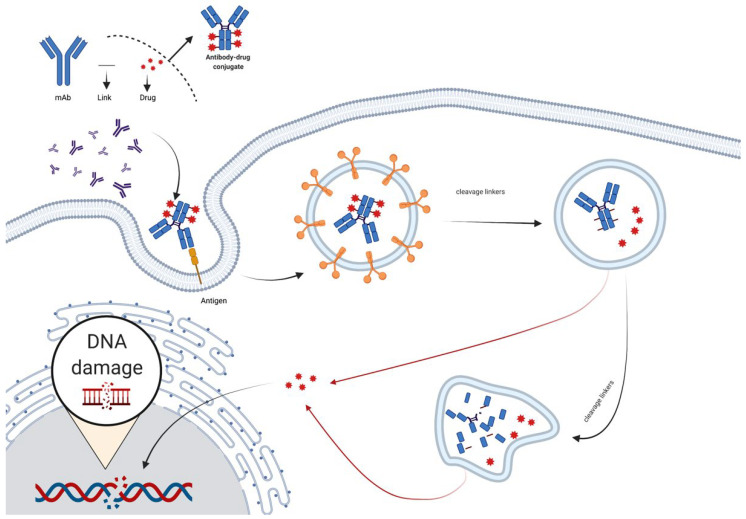
Pattern diagram of antibody–drug conjugates (ADC). Chemical linkers with labile bonds attach monoclonal antibodies to biologically active drugs. Internalized vesicles go through the endosome—lysosome pathway. Proteases digest the monoclonal antibody to release drugs (created with Biorender).

Because of the benefit of antibodies’ specific targeting functions and the disadvantage of chemo-drug systemic toxicity, antibody–drug conjugates were developed, which covalently linked mAbs with chemo drugs (Figure 5). ADCs improve drug tolerability while increasing cell killing selectivity. The flaws of ADC are also obvious; the unstable linkage between the drug and the antibody, as well as the low payloads of successful drug delivery to the targets, are two major concerns [17]. 

## 3. Infectious Diseases in Lung 

Lung infection can be broadly classified into the following subtypes: (1) Infectious asthma, which is caused by tracheal swelling caused by microbes. (2) Chronic obstructive pulmonary disease (COPD), which is caused by the inhalation of certain harmful particles and abnormal inflammatory response. (3) Pneumonia, which is caused by microbe contamination in the alveoli, SARS-CoV, SARS-CoV-2, MARS, and so on. (4) Tuberculosis, slowly progressive pneumonia caused by the bacterium *Mycobacterium tuberculosis*. (5) The flu. For this discussion, we selected several representative diseases from each category.

### 3.1. COVID-19

#### 3.1.1. The Application of Neutralizing Antibodies in Patients with COVID-19

The outbreak of coronavirus disease 2019 (COVID-19) has placed a great burden on global public health, with more than 170,000,000 persons infected, according to the WHO. Infected individuals suffer from fever, diarrhea, and labored breathing. Treatment and prevention methods are urgently needed to fight the COVID-19 pandemic.

Thanks to collaborative work by researchers worldwide, various types of vaccines have been invented [18]. These vaccines have thus far been proven safe, with no serious side effects reported yet [19]. The ultimate goal of vaccination is to generate specific antibodies corresponding to the severe acute respiratory syndrome-coronavirus 2 (SARS-CoV-2), using either the inactivated vaccine [20], live-attenuated vaccine [21], or the viral vector vaccine [22]. Among the various types of antibodies generated by the human immune system, there is the neutralizing antibody, which binds to a specific position on the virus surface and disables its infection ability. The binding of nanobodies to the SARS-CoV-2 receptor-binding domain (RBD) can prevent its reaction with angiotensin-converting enzyme 2 (ACE2) and subsequently stop the infection process [23]. In contrast, binding to ACE2 epitope has the same effect. Besides the chemically modified and manufactured neutralizing antibody vaccine, the human immune system itself can produce neutralizing antibodies. The application of convalescent plasma therapy in treating COVID-19 patients has proven effective under some specific circumstances [24].

#### 3.1.2. The Mechanisms and Targeting Sides for Neutralizing Antibodies in Treating Patients with COVID-19

Coronavirus 2 virions are embedded in a lipid bilayer with a protruding spike (S) protein on its surface [25]. The S protein structure is made up of two subunits, namely S1 and S2. SARS-CoV-2 enters somatic cells by attaching to ACE2. Initially, researchers suspected that the binding affinities of S protein with ACE2 are similar in SARS-CoV-2 and SARS-CoV. However, some recent studies indicated that the SARS-CoV-2 binding affinity is greater than that of SARS-CoV, which may explain why SARS-CoV-2 is more infectious and fatal. One of the unique attributes that distinguishes SARS-CoV-2 from SARS-CoV is a furin cleavage site at the frontier between the S1/S2 subunits [26]. The S protein can flexibly change its conformation [27]. Indeed, the fusion of viral and cellular membranes is driven by the extensive structural changes of the S protein [28]. With the use of Cryo-EM, the distribution and density of S protein were revealed. The interaction of virion and cell-only affects a small portion of spikes, indicating the potential of developing vaccines based on this target [29]. 

Neutralizing antibodies could protect against SARS-CoV-2 infection in several major ways. First, by targeting sites on the SARS-CoV-2 surface and disabling its infection ability. Second, neutralizing antibodies could target sites on somatic cells, thereby preventing the entry of SARS-CoV-2. Additionally, some neutralizing antibodies can enhance immune cell function and prevent anergy, while novel targets include viral uncoating, metabolism, and recognition. 

Besides the neutralizing antibody targeting SARS-CoV-2 RBD, the correlation between cytokines and COVID-19 is worth noting. Some COVID-19 patients have presented with cytokine storm, which can lead to acute respiratory distress syndrome and multiple organ dysfunction syndromes [30]. Cytokine storm of interleukin (IL)-2, IL-4, tumor necrosis factor-alpha (TNF-α), interferon-gamma (IFN-γ), and C-reactive protein has not been directly associated with COVID-19 [31], whereas IL-6 and IL-10 were found to be COVID-19 severity predictors [32]. These findings provide insights into inflammation control and the neutralization of antibody development. Maintaining a balance between immune activation and inflammation control is essential in this process.

#### 3.1.3. Approaches to the Identification of Neutralizing Antibodies for COVID-19

Due to the similarity between SARS-CoV and SARS-CoV-2, it is reasonable to screen out potential antibodies that can cross-react with SARS-CoV-2 [33]. Genome-wide analysis indicated more than 80% similarity between these two species. Although a significant portion of antibodies against SARS-CoV failed to neutralize SARS-CoV-2, the CR3022 antibody has proven effective in the treatment of patients with SARS-CoV-2 infection. This is attributed to its potent binding ability with SARS-CoV-2 RBD and the lack of epitope overlap with the ACE2 binding site [34]. H014, a kind of humanized monoclonal antibody, can neutralize both SARS-CoV-2 and SARS-CoV pseudo-viruses by binding to the RBD. Moreover, H014 treatment reduced SARS-CoV-2 titers in COVID-19-infected lungs in mice, with an associated improvement in pulmonary pathology [35].

In addition to the identification of neutralizing antibodies from SARS-CoV experience, potent neutralizing antibodies against SARS-CoV-2 have been identified in convalescent plasma. Even the milk from previously COVID-19-infected mothers has shown neutralizing effects against specific targets, including a SARS-CoV-2 pseudovirus and clinical isolate [36]. To investigate the efficacy of convalescent plasma in the treatment of patients with moderate COVID-19, an open-label phase-2 trial was conducted in India [37]. A total of 464 volunteers were recruited, with 235 assigned to convalescent plasma treatment and the rest to a standard care regimen. No improvement in prognosis or overall survival was found, although the result may have been compromised because the neutralizing antibody titers could not be measured at the time of the intervention. However, the results differed when a specific neutralizing antibody was extracted and applied at a high load. In a phase-2 trial involving 452 outpatients with mild or moderate COVID-19, a single intravenous infusion of the neutralizing antibody LY-CoV555, which targets the S protein and was extracted from a recovered SARS-CoV-2 patient, appeared to accelerate the natural decline in viral load [38]. 

#### 3.1.4. The Neutralizing Antibody Delivery Methods used in the Treatment of Patients with COVID-19

The delivery method for neutralizing antibodies varies. Apart from the protein delivery approach and the plasma infusion method, nanotechnologies have also attracted the attention of researchers. Drugs embedded in nanoparticles have proven more efficient in clinical practice than normal drug delivery methods. The nanoparticle vaccine, NVX-CoV2373, containing trimeric S protein and Matrix-M1 adjuvant, was declared safe and effective based on phase-1–2 trial results, having elicited an immune response exceeding than that seen in COVID-19 convalescent serum [39]. 

#### 3.1.5. The Efficacy of Neutralizing Antibody Compared with mRNA Vaccines in Treating Patients with COVID-19

Messenger RNA (mRNA) vaccines for COVID-19 prevention have been developed as well. The basic principle of the mRNA vaccine is similar to neutralizing antibody treatment. The body is injected with specific mRNA, which expresses specific proteins in somatic cells, prompting the immune system to generate corresponding antibodies. Pfizer claims that its Novavax vaccine is 90% effective; the efficacy of the Curevac vaccine was 47% [40]. In phase-3 trials, the mRNA-1273 vaccine, which encodes a SARS-CoV-2 spike-like protein, stabilized in the prefusion conformation, showed strong neutralizing antibody responses [41]. 

Although the theory behind mRNA is simple, its clinical application is highly challenging owing to the limitations to drug delivery. mRNAs are very labile and susceptible to degradation by nuclease or immune cells. These issues could be addressed by chemical modifications to improve the stability of mRNAs. Furthermore, advanced techniques have enabled the incorporation of larger mRNA molecules into a well-designed vector that can fuse itself with the plasma membrane [42]. Neutralizing antibodies have a more rapid effect than mRNA or other vaccines [43], which elicit an immune response to generate antibodies. Ke Wang et al. pointed out the relatively low expression of ACE2 in the lung. Therefore, the entrance of SARS-CoV-2 may exist in other pathways. They found an interaction between somatic cell receptor CD147 and spike protein. CD147 mediates the viral entrance through endocytosis [44]. A randomized phase-1 and an exploratory phase-2 trial to test the efficacy of meplazumab, a humanized monoclonal antibody anti-CD147 lgG2, for treating patients with COVID-19, indicated that meplazumab could significantly reduce the recovery time from 13 days to 3 days on average [45].

#### 3.1.6. The Limitations of Neutralizing Antibodies in Treating Patients with COVID-19

The titers of neutralizing antibodies also play a significant role. The low titer of neutralizing antibodies in recovered COVID-19 patients was the main reason for the failure of a recent convalescent plasma clinical trial [46]. It is also the reason for the failure of convalescent plasma therapy, i.e., the transfer of neutralizing antibody-containing plasma from recovered patients to patients in need. Convalescent plasma therapy has been used for the treatment of other viral infections such as SARS-CoV, Middle East Respiratory Syndrome (MERS), Ebola, and avian influenza A [47]. 

The shortcomings of neutralizing antibodies are apparent as well. The vaccine development process is much slower than that for standard vaccines. Many people have already been vaccinated in China, but no wide application of neutralizing antibody vaccines has been reported. Furthermore, mutations of the virus may disable the antibody vaccine. It is concerning that spike mutations have been discovered in different places worldwide [48]. Researchers have demonstrated that S proteins with mutations in RBD show resistance toward monoclonal antibodies or convalescent plasma. However, the number of S proteins that are resistant to neutralizing antibodies is relatively low. Therefore, the influence of S protein mutations is of lesser concern, as the resistance may be eliminated by using multiple neutralizing antibodies targeting different neutralizing epitopes [49]. Moreover, considering that vaccine development and production are commercial enterprises, the antibody vaccine will have limited value once the pandemic is over. 

The true differences between applying neutralizing antibodies and vaccines lie in the patients with severe symptoms, such as acute respiratory distress syndrome, where the neutralizing antibody treatment is more effective at preventing disease progression to a more severe stage. Clinical data showed that neutralizing antibodies were not effective in either patient with mild symptoms or patients with end-stage disease because the patients in the former situation could self-recover [50], whereas the patients in the latter situation had associated damage to multiple vital organs.

Patient treatment is often not uniform. Neutralizing antibodies may react with the vaccine, disable its function, and ultimately inhibit any immune response as the antibodies would be neutralized as well. 

### 3.2. Asthma

Inflammation of the lower or upper respiratory tract causes asthma. In general, the CD4+ T cell is regarded as the most important player in this disease. Furthermore, the Th2 subtype produces enough IL-4, IL-5, and IL-13 to cause inflammation [51]. At the same time, innate lymphoid cells group 2 (ILC2s) contribute to disease emergence by producing IL-5 and IL-13. Asthmatic patients frequently experience difficulty breathing and coughing. Non-atopic asthma and atopic asthma can be distinguished based on observable characteristics or phenotype. Asthma is classified into two types based on systems biology: T2-high and non-T2-high. The presence of airway eosinophilia is the basis for this well-established classification. Because cytokines are abundant in T2 asthma, advances in the treatment of T2 asthma by targeting cytokines have been made [52]. 

### 3.3. The Abnormally Altered Signaling Pathways in Patients with Asthma 

The intracellular signal changes that occur when cells are exposed to high levels of IL-4, IL-5, and IL-13 have been thoroughly studied [53]. The Janus kinase–signal transducer and activator of transcription (JAK-STAT pathway) were discovered to be involved in the pro-inflammatory process. TGF-, IL-1, IL-4 (JAK1/3-STAT3/5/6), IL-5 (JAK2-STAT3), IL-6, and IL-13 (JAK1) [54] are JAK-STAT pathway activators in asthma. JAK has the ability to phosphorylate STATs. STATs will form dimers that will travel into the nucleus and regulate the expression of cytokine-responsive genes.

### 3.4. The Application of Monoclonal Antibodies in Patients with T2 Asthma

Four commonly used mAbs in treating T2 asthma by reducing eosinophilic inflammation are mepolizumab, dupilumab, reslizumab, and benralizumab [55]. 

Mepolizumab (IgG1 kappa) inhibits IL-5 production. Because IL-5 is one of the most important cytokines initiating eosinophil growth and differentiation, using Mepolizumab results in a significant decrease in eosinophilia in the blood [56]. In a 2-year follow-up clinical trial [57], the use of mepolizumab significantly reduced the exacerbation rate (from 4.3 2.3 to 1.3 1.8; p 0.0001), as well as the need for oral corticosteroid doses and blood eosinophil count. The asthma control test score increased significantly (from 16.3 3.7 to 21.2 3.8; p 0.0001).

Reslizumab, similarly to mepolizumab, acts as an IL-5 antagonist. The application of this mAb, on the other hand, is unique. It must be injected intravenously rather than subcutaneously, and the volume of injection must be adjusted according to the patient’s weight. Reslizumab demonstrated excellent response in 58.6 percent of patients and clinically meaningful response in 16.3 percent of patients in a real-world test on 215 patients. [58]. 

Benralizumab combats the effect of over-activated IL-5 function by targeting the IL-5 receptor. It not only inhibits the function of the IL-5 receptor, but it can also direct the apoptosis process in eosinophils and basophils by recruiting NK cells [59]. Benralizumab application improved all patients’ situations at three months and six months treatment in a recently published multicenter study involving 42 patients. Furthermore, the number of oral corticosteroid cycles was significantly reduced [60].

Dupilumab (IgG4) works as an IL-4 receptor antagonist. It binds to the alpha chain of the IL-4 receptor [61]. In hematopoietic cells, the alpha chain of the IL-4 receptor binds with the c chain to form a type 1 IL-4 receptor, which only binds to IL-4. Furthermore, it can form a heterodimeric complex with the IL-13 receptor alpha chain one, which can be activated by both IL-4 and IL-13. Following that, the JAK-STAT pathway cascade will be activated as described above. Dupilumab works in two ways: first, it prevents IL-4 from binding to the IL-4 receptor alpha chain, and second, it may inhibit the recruitment of another IL-4 receptor alpha chain or the IL-13 receptor alpha 1 chain. In a clinical trial comparing dupilumab to placebo (724 patients), dupilumab significantly increased a variety of test scores (nasal polyp, patient-reported nasal congestion score, Lund–Mackay computed tomography scan score, peak nasal inspiratory flow, 22-item sinonasal outcome test score, all P.001). Furthermore, the side effect was insignificant enough to be overlooked.

### 3.5. The Possibility of Applying the Mabs Listed above to Treat Patients with COVID-19

Patients infected with SARS-CoV-2 would also be subjected to ferocious immune system activation. Because eosinophils play critical roles in the host’s defense system, IL-5 is in charge of activating eosinophils. As a result, cytokines are a good prognostic factor for COVID-19 patients. However, the patients will die as a result of dysregulated immune responses (cytokines storm). Corticosteroids, a commonly used inflammation control agent, were used in this study, but the inhibition of the immune system also slowed pathogen elimination, making its use paradoxical [62]. Nonetheless, the mAbs used to target specific cytokines or corresponding targets may be important. Interestingly and importantly, the administration of mepolizumab reduces eosinophils in the peripheral blood but has no effect on eosinophils in the airways. Furthermore, clinical evidence suggests that anti-IL-5 drugs are beneficial for patients with severe COVID-19. The explanation for such symptoms is largely due to an abnormal increase in neutrophils, eosinophils, and IL-5 levels, and mepolizumab can improve NK cell ability and B cell survival, thereby increasing anti-viral response [63].

## 4. Lung Cancer

Cancer and autoimmune disease can be compared to two sides of the same coin, with the former being caused in part by immune system downregulation and the latter being caused by immune reaction hyperactivation. Pathology classifies lung cancer into two subtypes: non-small cell lung cancer (lung squamous cell carcinoma, lung adenocarcinoma, and large cell carcinoma) and small cell lung cancer. Lung tumors exhibit nearly all of the hall markers described by D. Hanahan [64]. In general, growth signals (the majority of which play a significant role in embryo development) are abundantly expressed and provide a crushing momentum for antigrowth signaling. Meanwhile, angiogenesis and cancer metastasis-related signals are expressed in cancer tissue. In particular, epidermal growth factor receptor (EGFR) activation is common in non-small cell lung cancer, whereas the KIT receptor is the main player in small cell lung cancer [65]. Furthermore, because SCLCs have both autocrine and paracrine abilities, they can activate G-protein-coupled receptors with gastrin-releasing bombesin-like peptides. These activation cascades will have an impact on multiple subcellular signaling pathways [66]. Mutations are another feature of lung cancer, and some researchers believe they are far more important than abnormal gene expression [67]. The well-known TP53 gene mutation was found in 90% of SCLCs and 50% of NSCLCs, and MYC amplification was frequently found in SCLCs. 

### 4.1. The Mechanisms of Antibody Therapies in Treating Cancer

Neutralizing antibodies and monoclonal antibodies also interact with receptors on human somatic cells, which could be a key approach in cancer immunotherapy [68]. Antibodies that target immune cells also play an important role in immune cell hyper- and hypo-reactivity. In the field of lung cancer antibody therapy, immune checkpoints, classic or novel therapy targets on cancer cells, cytokines, and ADCs are the four main focus areas.

### 4.2. The Current Cytokine-Targeted Antibody Therapy and Mechanism 

Cytokines are small, secreted proteins that regulate immune cells and cancer cells by either inhibiting or promoting inflammation. Similarly, they can induce programmed death in abnormal cells or prolong the normal cell life. According to different classification methods, cytokines can be divided into several sub-groups. Cytokines secreted by lymphocytes and monocytes are called lymphokines and monokines, respectively. Furthermore, cytokines with chemotactic activities are defined as chemokines, whereas ILs are responsible for communication between leukocytes [69]. 

Various cytokines influence lymphocytes, which play a fundamental role in cytokine secretion by helper T cells (Th). Cytokines secreted by lymphocytes can influence other lymphocytes as well [70]. Four Th subtypes have been identified based on cytokine expression, namely T helper 1 (Th1), T helper 2 (Th2), T helper 17 (Th17), and T follicular helper (Tfh). Their developmental and regulatory pathways are different, as are their functions in immune responses. Th1 differentiation is regulated by IL-12 [71], whereas Th2 differentiation is mediated by IL-25/IL-33 [72]. In addition, Th17 differentiation is caused by the combined efforts of several different cytokines at different stages. These are transforming growth factor beta (TGF-β) [73], IL-6 [74], IL-21 [75], IL-23 [76], and IL-1 [77], respectively. Finally, Tfh differentiation is induced through transcription factors [78].

Previous studies evaluated the different functions of Th1 and Th2 based on the different cytokines they secreted. The number of Th1 cells is frequently higher in patients with autoimmune diseases, which means Th1 may cause inflammation. Indeed, the symptoms are caused by Th1 cells secreting proinflammatory factors, such as IFN-γ and lymphotoxin-α (Lt-α). Th2 has been recognized as a significant player in allergic diseases, such as allergic asthma. Th2 cells were found to regulate humoral immune response by producing cytokines such as IL-4, IL-5, IL-9, IL-10, and IL-13 [79]. The role of Th17 in cancer immunity is more complex. The role varies between different types of cancers—even the experimental settings have an effect. Both pro-tumorigenic and anti-tumorigenic effects have been observed [80]. This double-edged role of Th17 is attributed to the high level of IL-17 it produces. Besides, IL-17 expression is controlled by retinoic acid receptor-related orphan receptor gamma [81], and IL-17 is a critical proinflammatory cytokine in cancer [82]. Furthermore, Th17 cells recruit cytotoxic T lymphocytes (CTLs), natural killer (NK) cells, and neutrophils through the stimulation of IL-6 [83], IL-1β, and TNF-α, directly or indirectly via IL-17 [84].

One major source of concern is inflammation, which alters tumor cells as well as cells in the tumor microenvironment. Intriguingly and crucially, inflammation has both “good” and “bad” effects.

On the “positive” side, cytokines and chemokines produced by neutrophils and other innate immune cells cause immune cells to migrate into tumor tissue, where they can kill cancer cells. Some anti-inflammatory factors (IL-10, TGF-β) will balance this process at the same time. On the “bad” side, excessive inflammation causes metabolic and vascular changes, which are associated with a poor prognosis.

Some cytokine-targeting antibody drugs have been invented.

- Siltuximab. Because of a significant association between IL-6 staining and poor prognosis in an immunohistochemical analysis of ovarian cancer tissue, IL-6 was identified as a therapeutic target. A subsequent phase-2 clinical trial showed promising results for siltuximab, a monoclonal neutralizing antibody drug approved by the European Commission, in treating patients with platinum-resistant ovarian cancer [85]. However, a later clinical trial with siltuximab on patients with solid tumors (including non-small cell lung cancer) found no significant clinical activity [86]. A larger cohort of lung cancer patients should be recruited in order to determine the precise efficacy of siltuximab in lung cancer patients.
- Secukinumab IL-25, a subtype of IL-17, has been linked to a perplexing role in cancer. Although IL-25 inhibition therapy has shown promising results in cancer treatment, it, like IL-17, has a double-edged effect in cancer patients. On the one hand, IL-25 has been shown to increase the number of B cells and eosinophils in the tumor microenvironment, which is thought to be a tumor-suppressive function. IL-25, on the other hand, will initiate inflammatory cascades and type 2 immune responses. Thus, the administration of Virulizin and dihydrobenzofuran can increase IL-25 activity, whereas anti-IL-25 antibodies can inhibit it. However, either of these approaches has the potential to slow cancer growth [87]. Furthermore, IL-17 inhibitors such as secukinumab have already been shown to be effective [88]. In a case report, a patient with LUAD developed psoriasis as a result of the administration of pembrolizumab. The LUAD was successfully controlled after the co-administration of secukinumab and pembrolizumab [89].
- Canakinumab IL-1 promotes cancer metastasis by causing inflammation and a "hot" tumor microenvironment. IL-1 activation will also activate the PI3K-AKT pathway and, as a result, NF-κB. Canakinumab is a type of anti-IL-1 monoclonal antibody used in humans. In 2017, a double-blind clinical trial with 10061 patients was published [90]. These patients were cancer-free, but their high-sensitivity C-reactive protein level was elevated. Canakinumab also significantly reduced their levels of high-sensitivity C-reactive protein and IL-6. A recent trial also highlighted the importance of administering canakinumab to patients with various subtypes of lung cancer [91].
- Denosumab works as a RANKL (Receptor Activator of NF-κB) antagonist. RANKL was initially identified as a mediator of osteoclast function. Its function, however, is not limited to the bone but also includes the immune system as a dendritic cell activator and a role in tumor formation [92]. As a result, denosumab has the potential to reduce cancer bone metastasis. Denosumab significantly improved median overall survival in a phase-3 study of 702 patients with NSCLC, with normal (control group) side effects reported [93].
- ALT-803 works as an IL-15 superagonist. Because IL-15 inhibits tumor growth by activating NK cells and CD8+ T cells, intravenous administration of ALT-803 significantly increased NK cell numbers and had a minor effect on CD8+ T-cell expansion in patients with lung cancer [94]. The combination of ALT-803 and nivolumab has been shown to be safe and effective in lowering the refractory rate.

### 4.3. The Future Direction for Cytokine-Targeted Antibody Therapy

**Figure 6:**
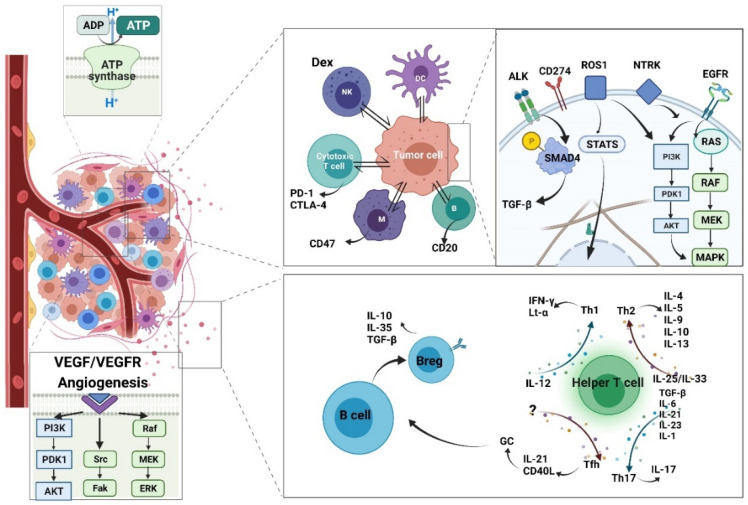
Multiple cytokines and corresponding cells are responsible for tumor eradication and immune suppression (created with BioRender).

The crosstalk between T cells and B cells is worth discussing. Tfh cells provide a bridge between CD4+ T cells and B cells by producing IL-21 and cluster of differentiation 40 ligand (CD40L) for development of the germinal center [95], where B cell hyperproliferation and hypermutation take place. It is not surprising that cancer-related research shows the presence of Tfh cells to be crucial in orchestrating a T-cell-dependent humoral immune response toward cancer cells. Compared with the many T cell subsets, the B cell subsets are minor, but their essential role in producing neutralizing antibodies cannot be ignored. Previous studies have identified several regulatory B cells (Bregs), which secrete cytokines with anti-inflammatory function, e.g., IL-10, IL-35, and TGF-β. The recently discovered IL-35-producing B cell (i35-Breg) also has a fundamental role in immunity and inflammation, as it can induce infectious tolerance itself, whereas conventional B cells need assistance from Th17, as mentioned above. The clinical application of i35-Bregs in autoimmune disease has been discussed. Furthermore, i35-Breg depletion may serve as a new therapeutic strategy against tumor cells [96] (Figure 6).

The development of more effective antibody-based drugs targeting IL-2 for lung disease is also worth discussing. Pérol L, et al. have already shown the existence of anti-IL-2 autoantibodies in non-obese diabetic mice and Type 1 Diabetes patients, and the IL-2 may serve as an autoimmune target for patients with Type 1 Diabetes [97]. This is not only because IL-2 serves a role in autoimmune disease [98,99], but also due to its regulatory role for Anti-inflammatory T regulatory cells (Treg) regulation, since IL-2 can control the immune response by maintaining Treg cell function and promoting immune response by stimulating classic T cells [100]. Importantly, the correlation between low survival and Treg infiltration in the tumor microenvironment has been well established. Therefore, the control of Treg through IL-2 and anti-IL-2 antibodies is considerable [101]. As data have accumulated, the advantages of computational tools in protein design have become evident. A computational approach to the design of neutralizing antibodies against natural cytokines is urgently needed. Fortunately, a recent study described a de novo computational approach to designing hyper-stable proteins, with higher affinity than natural cytokines toward IL [102]. This approach may find future clinical application. 

### 4.4. The Current Immune Checkpoint-Targeted Antibody Therapy and Mechanism

Besides the SARS-CoV-2 S protein, there are various other classic or novel therapeutic targets. Targeted immunotherapy is different from radiotherapy or chemotherapy. The latter methods influence tumor cell growth and survival directly, but will affect healthy cells as well, whereas immunotherapy boosts the immune response targeted at cancer cells. Under normal circumstances, cytotoxic CD8+ T cells can recognize antigens present on the cancer cell membrane, including overexpressed normal proteins and novel proteins. However, cancer may mimic normal cells by using negative feedback mechanisms, such as the inhibitory receptors, programmed cell death protein 1 (PD-1), and CTLA-4 [103]. PD-1 is an essential regulator of adaptive immune response and inhibitor of immune signaling. The receptor is presented on the T cell when the T cell receptor (TCR) complex is simulated or when specific receptors detect the cytokines. The combination of PD-1 and PD-1 receptors activates TCR/CD28 signaling and IL-2-dependent positive feedback, resulting in reduced cytokine production and cell cycle-related gene expression regulation. CTLA-4 blocking inhibits T cell functions by binding to CD80 and CD86. A CTLA-4 knockout experiment in mice showed a lymphoproliferative disorder since CTLA-4 elevates the T cell activation threshold, eliminating the immune response to some tumor antigens [104]. Anti-T-lymphocyte-associated-protein-4 (CTLA-4) and anti-PD-1 showed dramatic clinical outcomes [105], with significantly improved overall patient survival.

Some immune checkpoints targeting antibody drugs are listed below.

- Atezolizumab is a PD-L1 antagonist that is commonly used in patients with elevated PD-L1 protein levels but no EGFR or ALK mutation. It is also used in the treatment of patients with non-small cell lung cancer in conjunction with platinum chemotherapy or other functional antibodies (bevacizumab). Patients with small-cell lung cancer can also be treated with atezolizumab. In a clinical trial involving 572 NSCLC patients with high PD-L1 expression and wild-type EGFR and ALK, the Atezolizumab group had a significantly higher median overall survival than the chemotherapy group (7.1 months; *p* = 0.01) [106].
- Camrelizumab works as a PD-1 inhibitor. Camrelizumab, similarly to atezolizumab, appears to be ineffective in NSCLC patients with EGFR or ALK mutations. In a 146-patient clinical trial, the response rate and progression-free rate were both positively correlated with PD-L1 expression. It is a safe drug that is more effective than chemotherapy in NSCLC [107]. The combination of camrelizumab and anlotinib, along with the anlotinib dose, significantly increased the medium progression-free rate [108].
- Durvalumab is a PD-L1 inhibitor that is recommended for stage-III unresectable NSCLC. Durvalumab’s efficacy was evaluated in a 2017 PACIFIC trial, which enrolled 713 patients who were randomly assigned to either a placebo or a durvalumab group. This study yielded favorable PFS and OS outcomes [109]. It has been demonstrated in real-world settings that patients with higher PD-L1 expression may benefit more from durvalumab treatment. Unfortunately, autoimmune disease history and some comorbidities may prevent its use and reduce its effectiveness [110].

### 4.5. The Future Direction for Immune Checkpoints Therapy

The currently published research primarily focused on T cells, but macrophages and myeloid-derived cells are also involved. These adaptive and innate immune cells may all serve as potential targets. CD274 (PD-L1) is universally acknowledged as a potent immune checkpoint in T cells. Similarly, in macrophages, integrin-associated protein (CD47) is recognized as an immune checkpoint [111]. The integrin-associated protein (CD47)/signal-regulatory protein alpha (SIRPa)-axis is an example of a macrophage activator [112]. SIRPa is highly expressed on the surface of macrophages and other cells belonging to myeloid lineages, such as granulocytes, monocytes, and myeloid dendritic cells (DCs) [113]; CD47 is highly expressed in various cancer types. Transfused red blood cells [114] and bone marrow cells [115] from CD47–/– mice were rapidly eliminated from the circulation of wild-type mice. The ligation of CD47 and SIRPa activates inhibitory phosphatases, SHP-1 and SHP-2, along with the phosphorylation of intracellular immunoreceptor tyrosine-based inhibitory motifs, with resultant downregulation of immune activity, including the contractile engulfment ability of macrophages [116]. In summary, the activation of CD47 will induce a “do not eat me” signal, which can evade phagocytosis. Experiments in mice have verified the feasibility of therapy targeting the CD47/SIRPa axis, and clinical trials are underway. It is expected to have treatment value for both solid tumors and hematologic cancers [117].

B-lymphocyte antigen CD20 is also recognized as a neutralizing antibody target. CD20 is expressed on the B cell surface, serving as a marker for lymphocyte classification and a mediator of various intracellular signaling pathways. Rituximab is a chimeric anti-CD20 monoclonal antibody that directly mediates cytotoxicity and apoptosis. The anti-CD20 drug has already been recognized as an effective approach for hematological neoplasia treatment [118]. The combination of rituximab and chemotherapy (cyclophosphamide, doxorubicin, vincristine, and prednisone) has shown excellent results (Cerny et al. 2002). The direction of future immunotherapy research is becoming clearer as our understanding of the intracellular mechanism grows. 

However, side effects have been reported. Because the combination of PD-1 or CTLA-4 with corresponding receptors will prevent autoimmunity, the inhibition will cause the abnormal regulation of other pathways. Some of them may be other inhibitory receptors [119]. For example, TGN1412 (a CD28 superagonist antibody) caused a life-threatening cytokine release syndrome [120]. Therefore, the identification of a promising checkpoint inhibitor with negligible effects on other proteins is essential, as is the targeted delivery of immunotherapy drugs to the tumor.

### 4.6. Antibody Targets Membrane Protein and Sub-Cellular Signaling 

Besides the immune checkpoint strategy, there are novel therapies focusing on angiogenesis, which is an essential process for tumor cell survival and proliferation. These include the popular vascular endothelial growth factor (VEGF)/VEGF receptor signaling blockade drugs [121]. VEGF is essential for vascular homeostasis, tumor growth and metastasis, blinding eye diseases, and diabetic and hypertensive retinopathy [122,123]. Several decades ago, neutralizing anti-VEGF antibodies were reported to have promising inhibitory effects on solid tumor growth, ascites formation, and metastasis in mice. Humanized murine antibody A.4.6.1 enabled its use in clinical trials; it was eventually commercialized as bevacizumab [124]. Subsequently, a series of clinical trials were conducted in a wide range of tumors [125]. The development of neutralizing anti-VEGF antibodies has dramatically prolonged survival time in cancer patients, especially in patients with non-small cell lung cancer (NSCLC), glioblastoma, and ovarian cancer [126].

Roundabout guidance receptor 1 (ROBO1) is involved in angiogenesis as well. It was first associated with functions in axon guidance and neuronal precursor cell migration (provided by RefSeq, Mar 2009). Abnormal ROBO1 expression was found in ocular neovascular diseases [127]. Moreover, ROBO1 has been recognized as a tumor suppressor for various cancers [128], including breast cancer (BC). The ROBO1-neutralizing antibody, R5, reportedly had significant inhibitory effects on BC growth and metastasis. These effects were attributed to its effect on angiogenesis. R5 significantly reduced tubular formation of vascular plexi and human umbilical vein endothelial cell migration and tubular formation. These results suggest that R5 may be a promising drug for the treatment of BC by inhibiting tumor-induced angiogenesis [129].

Vasohibin 2 (VASH2) is a member of the vasohibin (VASH) family, related to the similar VASH1, which is mainly present in endothelial cells to inhibit angiogenesis. In contrast, VASH2 occurs mainly in cancer cells and has a stimulating effect on tumor growth. Peritoneal injection of anti-VASH2 neutralizing antibodies inhibited tumor growth and angiogenesis in a mouse xenograft model of human cancer cells [130]. These results suggest that anti-VASH2 neutralizing antibodies may serve as a new strategy for cancer treatment. However, neutralization of pro-angiogenic factors such as VEGFA has had limited clinical benefits, either because of the development of an adaptive circumvention of specific angiogenic blocks or because of intrinsic or pre-existing indifference [131]. 

### 4.7. Co-Administration with Chemo Drugs or Tyrosine Kinase Inhibitors 

Supposing that immunotherapy was not applicable to a patient’s individual situation, a chemotherapy agent would significantly change the TME, which may also cause unresponsiveness after multiple cycles. On the subject of urothelial bladder carcinomas, repopulation is caused by the proliferative response of cancer stem cells [132]. Prostaglandin E2 (PGE2) release promotes this process. Both PGE2-neutralizing antibodies and PGE2 signaling blockade drugs enhance the chemotherapeutic response of urothelial bladder carcinomas [133]. Therefore, applying neutralizing antibodies while treating patients with chemo drugs may be beneficial in treating lung cancers.

Meanwhile, the neutralization of pro-angiogenic factors will have a better prognosis if coupled with neutralizing antibodies or tyrosine kinase inhibitors targeting other signaling pathways or targets, such as epidermal growth factor receptor (EGFR), human epidermal growth factor receptor 2 (HER2), and anaplastic lymphoma receptor tyrosine kinase (ALK) [134]. Many targeted drugs for these targets were invented and put into clinical use. The majority of them received positive outcomes [135]. 

## 5. The Crosstalk between Treating Infectious Disease, Autoimmune Disease, and Cancer in the Lung

### 5.1. The Possibilities of Cross Applying Mab in Treating Infectious Disease and Cancers in the Lung 

In conclusion, neutralizing antibody therapy has promise, given that some important cytokines are secreted across a wide range of cancers. Some immunotherapy targets can also be found on a variety of tumors. Neutralizing antibodies, on the other hand, have yet to find widespread clinical application. Their safety and efficacy in cancer and COVID-19 treatment are still being debated. Furthermore, the structure and subsequent cascade pathway have not been determined, which has hampered the development of neutralizing antibody therapy. Furthermore, the rate of development cannot keep up with the spread of the COVID-19 pandemic or cancer. As a result, rapid development and manufacturing methods are critical.

However, things are a little different with monoclonal antibodies. There were many common pathological cytokines among different types of lung disease, particularly asthma and infectious disease. Following the theoretical examination and clinical trials, existing antibodies can be used to treat newly discovered infectious diseases. Cross administration, on the other hand, is difficult to implement in the immune checkpoint strategy because current immune checkpoint drugs are designed to improve T cell performance while inhibiting CD274. However, infectious diseases do not exhibit abnormal cell proliferation. Immunotherapy aimed at B cells could shed new light on the cross-use (Table 1). 

### 5.2. The Similarities in Immunity between Patients with Infectious Diseases and Cancers 

**Figure 7:**
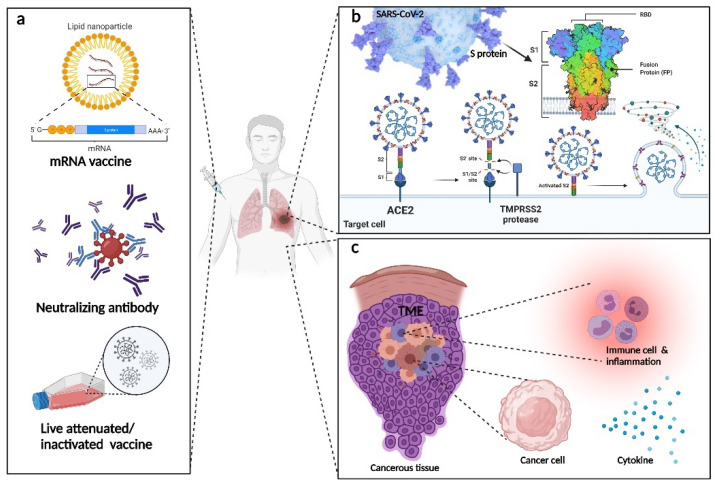
(**a**) Three common types of vaccines. (**b**) Diagrams showing COVID-19-infected somatic cells (**c**) and cancerous tissue, including the potential targets of neutralizing antibody therapy (Generated with BioRender).

It is universally acknowledged that both infectious disease and cancer weaken the immune system, either because of a pre-existing deficiency, the disease, or the treatment methods, while asthma enhances the immune response out of the normal range. Cancer can weaken the immune system by invading the bone marrow where immune cells are generated [136], e.g., in leukemia. However, other types of cancer, even somatic cancer cells, can metastasize to the bone marrow and consequently weaken the immune system. Meanwhile, some authors have reported that the cytokine secretion by cancer cells affects the tumor infiltration status. Tumors with high immune cell infiltration are defined as hot tumors, whereas tumors without strong immune responses are called cold tumors. High immune infiltration is associated with better tumor growth and metastasis [136]. Furthermore, both chemotherapy and radiotherapy are believed to have a significant side-effect on the patient’s immune system (Figure 7).

### 5.3. The Potential Signaling Pathway Can Be Targeted 

The cyclic GMP-AMP synthase (cGAS)–stimulator of interferon genes (STING) signaling pathway is involved in the radiotherapy cascade. It was found to regulate interferon secretion (e.g., type I interferon) and immune activation by sensing DNA double-strand breaks. The breaking of DNA double-strand by radiation is the basic principle of radiotherapy. Thus, the antitumor immunity will be enhanced [137]. However, when the radiation quantity rises out of a threshold (10–12 Gy), the interferon will simulate 3′-repair exonuclease 1 (TREX1), which subsequently down-regulates the cGAS–STING signaling pathway and degrades cytosolic double-stranded DNA [138].

Patients with immune deficiency or weakness usually have a worse prognosis. Although no radiotherapy was applied in patients with COVID-19, steroids were widely applied. Glucocorticosteroids effectively reduced mortality among infected patients and played an important role in sustaining patients who needed invasive mechanical ventilation [139]. Corticosteroid treatment tends to suppress the immune system, although there is an exception. A cytokine storm is characterized by the transformed activation of the immune system. This excessive elevation of circulating cytokines levels is associated with various infectious diseases, and symptomatic patients often present with vascular damage, immunopathology, and worsening clinical outcomes.

Cytokine storm has been observed in patients with COVID-19 and patients with cancer. Various drugs were developed to eliminate cytokine storm in patients with cancer; some proved effective even when applied to patients with COVID-19. Some tyrosine kinase inhibitors have been proven to be applicable. In the future, a monoclonal antibody with a similar function may be identified.

Acalabrutinib is used to treat patients with chronic lymphocytic leukemia. It is a small-molecule inhibitor targeting Bruton’s tyrosine kinase (BTK), a cytoplasmic tyrosine kinase that is essential in B-lymphocyte development, differentiation, and signaling [140].

In another study, using a macrophage-mediated model of atherosclerosis, bioinformatics results indicated that BTK is related to oxidative stress, ER stress, and inflammation, thus identifying BTK as a potential therapeutic target. Additional in silico analyses of expression values and protein–protein interactions also proved BTK to be a hub gene. Experimental verification proved the involvement of nuclear factor kappa B (NF-κB) signaling activation [141]. Acalabrutinib is also applicable to treating patients with COVID-19, as a recent study demonstrated that it reduced the oxygen requirements as well as the hospitalization rate and duration [142].

### 5.4. The Similarities of Surface Proteins Can Be Used as Biomarkers and Therapeutic Points

**Figure 8:**
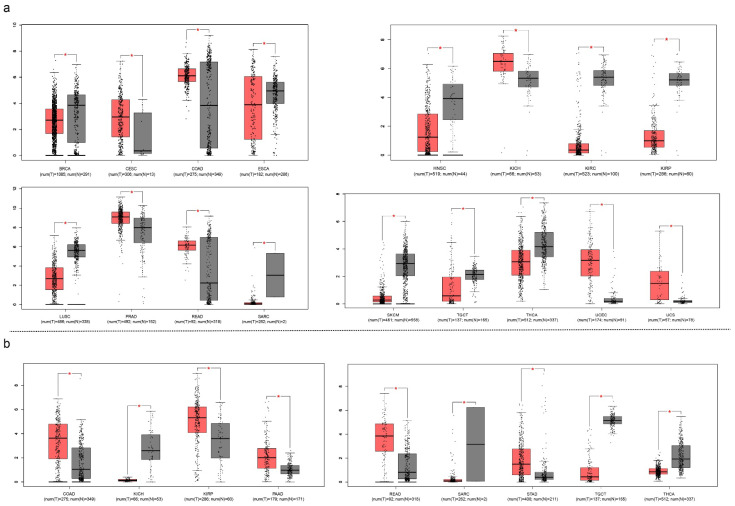
Expression of transmembrane serine protease 2 (TMPRSS2) (**a**) and angiotensin-converting enzyme 2 (ACE2) (**b**) across all types of cancers (only cancer types with a statistical difference were displayed). Asterisks represent levels of significance. * *p* < 0.05.

Another point of crosstalk between cancer and COVID-19 is their similar biomarkers. The function of SARS-CoV-2 involves a protein called transmembrane serine protease 2 (TMPRSS2), which has the ability to cleave S protein at the S29 site, making it essential to COVID-19 infection. However, it also identifies it as a potential therapeutic target. A knockdown experiment showed the essential role of TMPRSS2 in COVID-19-infected Calu-3 human airway epithelial cells [143]. TMPRSS2 is also one of the most advanced urine-based diagnostic and prognostic biomarkers for prostate cancer [144]. The activated TMPRSS2 releases its serine protease domain into the extracellular space. The activation of protease-activated receptor-2, a type of G protein, triggers a cascade that controls androgen-induced prostate cancer cell invasion and growth and may cause prostate cancer metastasis as well [145]. Its differential expression was observed in the following cancer types from the cancer genome atlas (TCGA) classification system: breast cancer (BRCA), cervical squamous cell carcinoma and endocervical adenocarcinoma (CESC), colon adenocarcinoma (COAD), esophageal carcinoma (ESCA), head-and-neck squamous cell carcinoma (HNSC), kidney chromophobe (KICH), kidney renal clear cell carcinoma (KIRC), kidney renal papillary cell carcinoma (KIRP), lung squamous cell carcinoma (LUSC), prostate adenocarcinoma (PRAD), rectum adenocarcinoma (READ), sarcoma (SARC), skin cutaneous melanoma (SKCM), testicular germ cell tumors (TGCT), thyroid carcinoma (THCA), uterine corpus endometrial carcinoma (UCEC), and uterine carcinosarcoma (UCS). The androgen receptor is not only used for the treatment of prostate cancer but is also found on lung cells [146]. We summarized the TMPRSS2 expression status across 33 types of cancer. Seventeen types of cancer showed differential expression between tumor tissue and normal tissue (Figure 8).

As previously mentioned, ACE2 mediates the recognition and entry of SARS-CoV-2 into somatic cells. Surprisingly, several types of malignant tumors have high ACE2 expression levels based on gene expression profiling interactive analysis (GEPIA) results [147]. The ACE2 protein was highly expressed in COAD, KICH, KIRP, pancreatic adenocarcinoma (PAAD), READ, SARC, stomach adenocarcinoma (STAD), TGCT, THCA, lung adenocarcinoma (LUAD), and LUSC, which suggested a high susceptibility of patients with these types of cancers to SARS-CoV-2 infection. A recent study confirmed this by reporting that COVID-19 patients with lung cancer have a higher risk of severe outcomes than that of patients without cancer [148].

Another notable point is that cells expressing low ACE2, such as immune cells, can also be potentially infected by SARS-CoV-2, as was shown for SARS-CoV, suggesting that other receptors may facilitate virus entry [149]. CD147, CD26, and cyclophilins were identified as supporting evidence for this hypothesis, as they were expressed in both epithelium and in immune cells. The expression of these receptors is related to age, gender, obesity, smoking, and disease status. This may explain the incidence rate and severity of COVID-19 in the population mentioned above.

In conclusion, patients with COVID-19 and patients with cancer, especially lung cancer, have many similar symptoms. Multiple identical significant cytokines and signaling pathways were identified in both COVID-19-infected and cancer tissues. Even some genetic similarities were found. Therefore, although some cancer drugs may be applicable to patients with COVID-19 as well, there are still many crosstalk identities between COVID-19 and cancer that remain to be discovered.

### 5.5. The Bioinformatics in Monoclonal Antibody Building and Target Identification

The bioinformatics approach should be followed in the development of monoclonal antibodies. The immune-informatics approach has successfully identified CTL and B cell epitopes in the SARS-CoV-2 surface glycoprotein [150]. Using a molecular dynamics approach, the interactions between identified epitopes and corresponding major histocompatibility complex (MHC) class I supertype representatives were reported. These identifications may serve as vaccine candidates. The bioinformatics approach would certainly accelerate the vaccine invention process; it has already been widely used in cancer biology. Numerous biomarkers and significant pathways have been identified using this approach, thus providing novel insights into cancer treatment and mechanism research.

Despite the rapid development of computer technology, it still cannot simulate all the chemical reactions or biological processes in cells, much less in an organ or entire human organism. The final evaluation needs to be performed in cell culture systems and animal models, followed by multiple phases of clinical trials. Building a reliable model is the highest priority in bioinformatics result verification [151]. There are different kinds of model animals, such as mice, hamsters, ferrets, and rabbits, each with its own advantages and shortcomings. The model should be chosen according to the experiment settings. Some conserved gene knockout models (e.g., ACE2) are relatively easy to build, while some complex genotype models are more challenging to build. Successful results in these model animals would permit the progression to clinical trials. 

Thanks to the development of high-throughput sequence technology and the advances in bioinformatics tools, it is possible to select agonist antibodies based on the affinity between ligand and receptor in the antibody’s activity. Moreover, Rosetta, AlphaFold, and other computational tools made it possible to predict the protein structure, which is useful in identifying potential antibodies.

Despite being a relatively new subject area, bioinformatics has already made many contributions to the biomedical field. Applying bioinformatics in potential target selection, corresponding neutralizing antibody design, and efficacy simulation, would aid in the successful application of neutralizing antibodies in cancer therapy. 

Nowadays, with the development of modern molecular and cellular biology, the overall structure and underlying mechanism of cancer or SARS-CoV-2 have been discussed extensively. However, many issues remain unexplored. Previous limitations prevented the development of effective treatment approaches, but the overall survival and prognosis of patients may be significantly improved through continued global collaborative efforts.

## 6. Preparing for Disease X in the Lung

Considering the variants of SARS-CoV-2 and some cancer cells, it is questionable whether these antibodies will remain applicable. The SARS-CoV-2 Delta variant has already shown reduced sensitivity toward neutralizing antibodies [152]. Thus, it would be desirable for neutralizing antibodies to target stable positions. Although it is not a good idea to focus on SARS-CoV-2 and cancer cells themselves, novel therapeutic targets for antibodies could also include the immune cell and microenvironment involved in the infectious or cancerous process, especially some cytokines. Meanwhile, the antibodies’ target surface proteins may not have a broader effect on different types of disease even in the same organ, but the antibodies’ target classic signaling pathways and cytokines may demonstrate cross use in different types of diseases. Herein, the antibodies for critical cytokines should be subject to more research in the future. 

The current review demonstrates that antibodies may be useful in the treatment of infectious diseases and lung cancer. The efficacies, side effects, and prognosis rates of antibodies vary depending on the mechanisms by which they enhance the immune response against cancer or infections. We should prepare for disease X [153] in the lung, which is not limited to the currently existing lung diseases. COVID-19 is not the first pathogen to have an impact on human health, and it will not be the last. Importantly, when a new disease, particularly an infectious disease, strikes a human, it is critical to use existing drugs for emergency use.

## Figures and Tables

**Table 1:** This is a summarized table describing the antibody drugs in lung disease *.

Disease	Subtypes	Target	Name
Lung cancer	NSCLC	VEGF	Bevacizumab
	NSCLC	EGFR	Necitumumab
	NSCLC, SCLC	PD-1	Nivolumab
	NSCLC	CD20	Rituximab
	NSCLC	PDCD1	Sintilimab
Lung infection	Asthma	IgE	Omalizumab
	Asthma	IL-4Rα	Dupilumab
	Asthma	IL-5	Mepolizumab
	COVID-19	Spike protein	Casirivimab
	COVID-19	IL-6	Tocilizumab
	COVID-19	GM-CSF	Lenzilumab
	COVID-19	IL-1β	Canakinumab
	COVID-19	VEGF	Bevacizumab
Autoimmune disease, may affect lung	Rheumatoid arthritis	TNFR-Fc	Etanercept
	Rheumarthritis	TNFa	Adalimumab
	Rheumatoid arthritis	IL-6	Tocilizumab
	SLE	BLyS	Belimumab

* Some drugs for COVID-19 are being tested.

## Data Availability

The GEPIA (http://gepia.cancer-pku.cn/) was accessed on 18 July 2021.

## Abbreviations

ACE2	Angiotensin-converting enzyme 2
COVID-19	Coronavirus disease 2019
DC	Dendritic cell
EV	Extracellular vesicle
IFN-γ	Interferon gamma
mRNA	Messenger RNA
NSCLC	Non-small cell lung cancer
PD-1	Programmed cell death protein 1
RBD	Receptor-binding domain
SARS-CoV-2	Severe acute respiratory syndrome-coronavirus 2
Th helper	T-cell
TMPRSS2	Transmembrane serine protease 2
VASH	Vasohibin
VEGF	Vascular endothelial growth factor
ACC	Adrenocortical carcinoma
BLCA	Bladder Urothelial Carcinoma
BRCA	Breast-invasive carcinoma
BC	Breast carcinoma
CESC	Cervical squamous cell carcinoma and endocervical adenocarcinoma
CHOL	Cholangiocarcinoma
COAD	Colon adenocarcinoma
COAD	Colon adenocarcinoma
READ	Rectum adenocarcinoma esophageal carcinoma
DLBC	Lymphoid Neoplasm Diffuse Large B-cell Lymphoma
ESCA	Esophageal carcinoma
GBM	Glioblastoma multiforme
GIST	Gastrointestinal Stromal Tumors
GC	Gastric Cancer
LGG	Low-Grade Glioma
HNSC	Head and Neck squamous cell carcinoma
KICH	Kidney Chromophobe
KC	Kidney Cancer
KIRC	Kidney renal clear cell carcinoma
KIRP	Kidney renal papillary cell carcinoma
LAML	Acute Myeloid Leukemia
LC	Lymphocyte cancer
LGG	Brain Lower-Grade Glioma
LIHC	Liver hepatocellular carcinoma
LUAD	Lung adenocarcinoma
LUSC	Lung squamous cell carcinoma
MESO	Mesothelioma
MM	Multiple Myeloma
OV	Ovarian serous cystadenocarcinoma
PAAD	Pancreatic adenocarcinoma
PNET	Pancreatic neuroendocrine tumor
PCPG	Pheochromocytoma and Paraganglioma
PRAD	Prostate adenocarcinoma
READ	Rectum adenocarcinoma
RCC	Renal cell carcinoma
SARC	Sarcoma
SKCM	Skin Cutaneous Melanoma
STAD	Stomach adenocarcinoma
STES	Stomach and Esophageal carcinoma
TGCT	Testicular Germ Cell Tumors
THCA	Thyroid carcinoma
THYM	Thymoma
UCEC	Uterine Corpus Endometrial Carcinoma
UCS	Uterine Carcinosarcoma
UVM	Uveal Melanoma

## References

[1] Schroeder H.W., Cavacini L. (2010). Structure and function of immunoglobulins. J. Allergy Clin. Immunol..

[2] Jasemi S., Erre G.L., Cadoni M.L., Bo M., Sechi L.A. (2021). Humoral Response to Microbial Biomarkers in Rheumatoid Arthritis Patients. J. Clin. Med..

[3] Bo M., Erre G.L., Niegowska M., Piras M., Taras L., Longu M.G., Passiu G., Sechi L.A. (2018). Interferon regulatory factor 5 is a potential target of autoimmune response triggered by Epstein-barr virus and Mycobacterium avium subsp. paratuberculosis in rheumatoid arthritis: Investigating a mechanism of molecular mimicry. Clin. Exp. Rheumatol..

[4] Bo M., Niegowska M., Eames H.L., Almuttaqi H., Arru G., Erre G.L., Passiu G., Khoyratty T.E., van Grinsven E., Udalova I.A. (2020). Antibody response to homologous epitopes of Epstein-Barr virus, Mycobacterium avium subsp. paratuberculosis and IRF5 in patients with different connective tissue diseases and in mouse model of antigen-induced arthritis. J. Transl. Autoimmun..

[5] Schittny J.C. (2017). Development of the lung. Cell Tissue Res..

[6] Janeway C.A., Travers P., Walport M. (2007). Immunobiology.

[7] Wachtman L., Mansfield K., Abee C.R., Mansfield K., Tardif S., Morris T. (2012). Chapter 1—Viral Diseases of Nonhuman Primates. Nonhuman Primates in Biomedical Research.

[8] Cantuti-Castelvetri L., Ojha R., Pedro L.D., Djannatian M., Franz J., Kuivanen S., van der Meer F., Kallio K., Kaya T., Anastasina M. (2020). Neuropilin-1 facilitates SARS-CoV-2 cell entry and infectivity. Science.

[9] Wang X., Ren J., Gao Q., Hu Z., Sun Y., Li X., Rowlands D.J., Yin W., Wang J., Stuart D.I. (2015). Hepatitis A virus and the origins of picornaviruses. Nature.

[10] Burdick R.C., Li C., Munshi M., Rawson J.M.O., Nagashima K., Hu W.S., Pathak V.K. (2020). HIV-1 uncoats in the nucleus near sites of integration. Proc. Natl. Acad. Sci. USA.

[11] Zhang M., Wang Y., He W., Sun Y., Guo Y., Zhong W., Gao Q., Liao M., Wang X., Cai Y. (2020). Design, Synthesis, and Evaluation of Novel Enterovirus 71 Inhibitors as Therapeutic Drug Leads for the Treatment of Human Hand, Foot, and Mouth Disease. J. Med. Chem..

[12] Dubel S. (2010). Handbook of Therapeutic Antibodies: Technologies, Emerging Developments and Approved Therapeutics.

[13] Synnestvedt M.B., Chen C., Holmes J.H. (2005). CiteSpace II: Visualization and knowledge discovery in bibliographic databases. AMIA. Annu. Symp. Proc. AMIA Symp..

[14] Ochoa M.C., Minute L., Rodriguez I., Garasa S., Perez-Ruiz E., Inogés S., Melero I., Berraondo P. (2017). Antibody-dependent cell cytotoxicity: Immunotherapy strategies enhancing effector NK cells. Immunol. Cell Biol..

[15] Nie M., Feng B., Liu C., Tu Y., Chen X., Wu F. (2021). Production and characterization of polyclonal and monoclonal antibodies of lamprey pore-forming protein. Protein Expr. Purif..

[16] Hendriks D., Choi G., de Bruyn M., Wiersma V.R., Bremer E., Galluzzi L. (2017). Chapter Seven—Antibody-Based Cancer Therapy: Successful Agents and Novel Approaches. International Review of Cell and Molecular Biology.

[17] Nejadmoghaddam M.R., Minai-Tehrani A., Ghahremanzadeh R., Mahmoudi M., Dinarvand R., Zarnani A.H. (2019). Antibody-Drug Conjugates: Possibilities and Challenges. Avicenna J. Med. Biotechnol..

[18] Poland G.A., Ovsyannikova I.G., Kennedy R.B. (2020). SARS-CoV-2 immunity: Review and applications to phase 3 vaccine candidates. Lancet.

[19] Castells M.C., Phillips E.J. (2021). Maintaining Safety with SARS-CoV-2 Vaccines. N. Engl. J. Med..

[20] Xia S., Duan K., Zhang Y., Zhao D., Zhang H., Xie Z., Li X., Peng C., Zhang Y., Zhang W. (2020). Effect of an Inactivated Vaccine Against SARS-CoV-2 on Safety and Immunogenicity Outcomes: Interim Analysis of 2 Randomized Clinical Trials. JAMA.

[21] Karpiński T.M., Ożarowski M., Seremak-Mrozikiewicz A., Wolski H., Wlodkowic D. (2021). The 2020 race towards SARS-CoV-2 specific vaccines. Theranostics.

[22] Logunov D.Y., Dolzhikova I.V., Shcheblyakov D.V., Tukhvatulin A.I., Zubkova O.V., Dzharullaeva A.S., Kovyrshina A.V., Lubenets N.L., Grousova D.M., Erokhova A.S. (2021). Safety and efficacy of an rAd26 and rAd5 vector-based heterologous prime-boost COVID-19 vaccine: An interim analysis of a randomised controlled phase 3 trial in Russia. Lancet.

[23] Huo J., Le Bas A., Ruza R.R., Duyvesteyn H.M.E., Mikolajek H., Malinauskas T., Tan T.K., Rijal P., Dumoux M., Ward P.N. (2020). Neutralizing nanobodies bind SARS-CoV-2 spike RBD and block interaction with ACE2. Nat. Struct. Mol. Biol..

[24] Duan K., Liu B., Li C., Zhang H., Yu T., Qu J., Zhou M., Chen L., Meng S., Hu Y. (2020). Effectiveness of convalescent plasma therapy in severe COVID-19 patients. Proc. Natl. Acad. Sci. USA.

[25] Neuman B.W., Buchmeier M.J. (2016). Supramolecular Architecture of the Coronavirus Particle. Adv. Virus Res..

[26] Walls A.C., Park Y.-J., Tortorici M.A., Wall A., McGuire A.T., Veesler D. (2020). Structure, Function, and Antigenicity of the SARS-CoV-2 Spike Glycoprotein. Cell.

[27] Zhang J., Cai Y., Xiao T., Lu J., Peng H., Sterling S.M., Walsh R.M., Rits-Volloch S., Zhu H., Woosley A.N. (2021). Structural impact on SARS-CoV-2 spike protein by D614G substitution. Science.

[28] Cai Y., Zhang J., Xiao T., Peng H., Sterling S.M., Walsh R.M., Rawson S., Rits-Volloch S., Chen B. (2020). Distinct conformational states of SARS-CoV-2 spike protein. Science.

[29] Ke Z., Oton J., Qu K., Cortese M., Zila V., McKeane L., Nakane T., Zivanov J., Neufeldt C.J., Cerikan B. (2020). Structures and distributions of SARS-CoV-2 spike proteins on intact virions. Nature.

[30] Li H.P., He X., Zhang L., Li C.X., Li S.Q., Li Q.Y. (2021). Therapeutic Agents Rounding Up the Immunopathology of COVID-19. Ther. Clin. Risk Manag..

[31] Luo W., Zhang J.W., Zhang W., Lin Y.L., Wang Q. (2021). Circulating levels of IL-2, IL-4, TNF-α, IFN-γ, and C-reactive protein are not associated with severity of COVID-19 symptoms. J Med. Virol..

[32] Han H., Ma Q., Li C., Liu R., Zhao L., Wang W., Zhang P., Liu X., Gao G., Liu F. (2020). Profiling serum cytokines in COVID-19 patients reveals IL-6 and IL-10 are disease severity predictors. Emerg. Microbes Infect..

[33] Zhu Z., Chakraborti S., He Y., Roberts A., Sheahan T., Xiao X., Hensley L.E., Prabakaran P., Rockx B., Sidorov I.A. (2007). Potent cross-reactive neutralization of SARS coronavirus isolates by human monoclonal antibodies. Proc. Natl. Acad. Sci. USA.

[34] Tian X., Li C., Huang A., Xia S., Lu S., Shi Z., Lu L., Jiang S., Yang Z., Wu Y. (2020). Potent binding of 2019 novel coronavirus spike protein by a SARS coronavirus-specific human monoclonal antibody. Emerg. Microbes Infect..

[35] Lv Z., Deng Y.-Q., Ye Q., Cao L., Sun C.-Y., Fan C., Huang W., Sun S., Sun Y., Zhu L. (2020). Structural basis for neutralization of SARS-CoV-2 and SARS-CoV by a potent therapeutic antibody. Science.

[36] van Keulen B.J., Romijn M., Bondt A., Dingess K.A., Kontopodi E., van der Straten K., den Boer M.A., Burger J.A., Poniman M., Bosch B.J. (2021). Human Milk from Previously COVID-19-Infected Mothers: The Effect of Pasteurization on Specific Antibodies and Neutralization Capacity. Nutrients.

[37] Agarwal A., Mukherjee A., Kumar G., Chatterjee P., Bhatnagar T., Malhotra P. (2020). Convalescent plasma in the management of moderate COVID-19 in adults in India: Open label phase II multicentre randomised controlled trial (PLACID Trial). BMJ Br. Med. J..

[38] Chen P., Nirula A., Heller B., Gottlieb R.L., Boscia J., Morris J., Huhn G., Cardona J., Mocherla B., Stosor V. (2021). SARS-CoV-2 Neutralizing Antibody LY-CoV555 in Outpatients with COVID-19. N. Engl. J. Med..

[39] Keech C., Albert G., Cho I., Robertson A., Reed P., Neal S., Plested J.S., Zhu M., Cloney-Clark S., Zhou H. (2020). Phase 1-2 Trial of a SARS-CoV-2 Recombinant Spike Protein Nanoparticle Vaccine. N. Engl. J. Med..

[40] Callaway E. (2020). What Pfizer’s landmark COVID vaccine results mean for the pandemic. Nature.

[41] Corbett K.S., Edwards D.K., Leist S.R., Abiona O.M., Boyoglu-Barnum S., Gillespie R.A., Himansu S., Schafer A., Ziwawo C.T., DiPiazza A.T. (2020). SARS-CoV-2 mRNA vaccine design enabled by prototype pathogen preparedness. Nature.

[42] Wadhwa A., Aljabbari A., Lokras A., Foged C., Thakur A. (2020). Opportunities and Challenges in the Delivery of mRNA-based Vaccines. Pharmaceutics.

[43] Jackson L.A., Anderson E.J., Rouphael N.G., Roberts P.C., Makhene M., Coler R.N., McCullough M.P., Chappell J.D., Denison M.R., Stevens L.J. (2020). An mRNA Vaccine against SARS-CoV-2-Preliminary Report. N. Engl. J. Med..

[44] Wang K., Chen W., Zhang Z., Deng Y., Lian J.Q., Du P., Wei D., Zhang Y., Sun X.X., Gong L. (2020). CD147-spike protein is a novel route for SARS-CoV-2 infection to host cells. Signal Transduct. Target. Ther..

[45] Bian H., Zheng Z.H., Wei D., Wen A., Zhang Z., Lian J.Q., Kang W.Z., Hao C.Q., Wang J., Xie R.H. (2021). Safety and efficacy of meplazumab in healthy volunteers and COVID-19 patients: A randomized phase 1 and an exploratory phase 2 trial. Signal Transduct. Target. Ther..

[46] Larsen S.E., Berube B.J., Pecor T., Cross E., Brown B.P., Williams B., Johnson E., Qu P., Carter L., Wrenn S. (2021). Qualification of ELISA and neutralization methodologies to measure SARS-CoV-2 humoral immunity using human clinical samples. Biorxiv Prepr. Serv. Biol..

[47] Shen C., Wang Z., Zhao F., Yang Y., Li J., Yuan J., Wang F., Li D., Yang M., Xing L. (2020). Treatment of 5 Critically Ill Patients With COVID-19 With Convalescent Plasma. JAMA.

[48] Winger A., Caspari T. (2021). The Spike of Concern-The Novel Variants of SARS-CoV-2. Viruses.

[49] Weisblum Y., Schmidt F., Zhang F., DaSilva J., Poston D., Lorenzi J.C., Muecksch F., Rutkowska M., Hoffmann H.H., Michailidis E. (2020). Escape from neutralizing antibodies by SARS-CoV-2 spike protein variants. Elife.

[50] Zeng Q.L., Yu Z.J., Gou J.J., Li G.M., Ma S.H., Zhang G.F., Xu J.H., Lin W.B., Cui G.L., Zhang M.M. (2020). Effect of Convalescent Plasma Therapy on Viral Shedding and Survival in Patients With Coronavirus Disease 2019. J. Infect. Dis..

[51] Wenzel S.E., Schwartz L.B., Langmack E.L., Halliday J.L., Trudeau J.B., Gibbs R.L., Chu H.W. (1999). Evidence that severe asthma can be divided pathologically into two inflammatory subtypes with distinct physiologic and clinical characteristics. Am. J. Respir. Crit. Care Med..

[52] Kuruvilla M.E., Lee F.E., Lee G.B. (2019). Understanding Asthma Phenotypes, Endotypes, and Mechanisms of Disease. Clin. Rev. Allergy Immunol..

[53] Salas A., Hernandez-Rocha C., Duijvestein M., Faubion W., McGovern D., Vermeire S., Vetrano S., Vande Casteele N. (2020). JAK-STAT pathway targeting for the treatment of inflammatory bowel disease. Nat. Rev. Gastroenterol. Hepatol..

[54] Bao L., Zhang H., Chan L.S. (2013). The involvement of the JAK-STAT signaling pathway in chronic inflammatory skin disease atopic dermatitis. JAK-STAT.

[55] Matucci A., Vivarelli E., Nencini F., Maggi E., Vultaggio A. (2021). Strategies Targeting Type 2 Inflammation: From Monoclonal Antibodies to JAK-Inhibitors. Biomedicines.

[56] Cada D.J., Bindler R.J., Baker D.E. (2016). Mepolizumab. Hosp. Pharm..

[57] Kallieri M., Zervas E., Katsoulis K., Fouka E., Porpodis K., Samitas K., Papaioannou A.I., Kipourou M., Gaki E., Vittorakis S. (2020). Mepolizumab in Severe Eosinophilic Asthma: A 2-Year Follow-Up in Specialized Asthma Clinics in Greece: An Interim Analysis. Int. Arch. Allergy Immunol..

[58] Wechsler M.E., Peters S.P., Hill T.D., Ariely R., DePietro M.R., Driessen M.T., Terasawa E.L., Thomason D.R., Panettieri R.A. (2021). Clinical Outcomes and Health-Care Resource Use Associated With Reslizumab Treatment in Adults With Severe Eosinophilic Asthma in Real-World Practice. Chest.

[59] Bakakos A., Rovina N., Bakakos P. (2021). Treatment Challenges in Severe Eosinophilic Asthma: Differential Response to Anti-IL-5 and Anti-IL-5R Therapy. Int. J. Mol. Sci..

[60] Padilla-Galo A., Levy-Abitbol R., Olveira C., Valencia Azcona B., Pérez Morales M., Rivas-Ruiz F., Tortajada-Goitia B., Moya-Carmona I., Levy-Naon A. (2020). Real-life experience with benralizumab during 6 months. BMC Pulm. Med..

[61] Harb H., Chatila T.A. (2020). Mechanisms of Dupilumab. Clin. Exp. Allergy J. Br. Soc. Allergy Clin. Immunol..

[62] Russell C.D., Millar J.E., Baillie J.K. (2020). Clinical evidence does not support corticosteroid treatment for 2019-nCoV lung injury. Lancet.

[63] Pala D., Pistis M. (2021). Anti-IL5 Drugs in COVID-19 Patients: Role of Eosinophils in SARS-CoV-2-Induced Immunopathology. Front. Pharm..

[64] Hanahan D., Weinberg R.A. (2000). The hallmarks of cancer. Cell.

[65] Imyanitov E.N., Kuligina E.S., Belogubova E.V., Togo A.V., Hanson K.P. (2005). Mechanisms of lung cancer. Drug Discov. Today Dis. Mech..

[66] Forgacs E., Zöchbauer-Müller S., Oláh E., Minna J.D. (2001). Molecular genetic abnormalities in the pathogenesis of human lung cancer. Pathol. Oncol. Res. POR.

[67] Stephens P., Hunter C., Bignell G., Edkins S., Davies H., Teague J., Stevens C., O’Meara S., Smith R., Parker A. (2004). Lung cancer: Intragenic ERBB2 kinase mutations in tumours. Nature.

[68] Payne S., Payne S. (2017). Chapter 6—Immunity and Resistance to Viruses. Viruses.

[69] Zhang J.M., An J. (2007). Cytokines, inflammation, and pain. Int. Anesthesiol. Clin..

[70] Lund F.E. (2008). Cytokine-producing B lymphocytes-key regulators of immunity. Curr. Opin. Immunol..

[71] Trinchieri G. (2003). Interleukin-12 and the regulation of innate resistance and adaptive immunity. Nat. Rev. Immunol..

[72] Cayrol C., Duval A., Schmitt P., Roga S., Camus M., Stella A., Burlet-Schiltz O., Gonzalez-de-Peredo A., Girard J.P. (2018). Environmental allergens induce allergic inflammation through proteolytic maturation of IL-33. Nat. Immunol..

[73] Mangan P.R., Harrington L.E., O’Quinn D.B., Helms W.S., Bullard D.C., Elson C.O., Hatton R.D., Wahl S.M., Schoeb T.R., Weaver C.T. (2006). Transforming growth factor-beta induces development of the T(H)17 lineage. Nature.

[74] Bettelli E., Carrier Y., Gao W., Korn T., Strom T.B., Oukka M., Weiner H.L., Kuchroo V.K. (2006). Reciprocal developmental pathways for the generation of pathogenic effector TH17 and regulatory T cells. Nature.

[75] Nurieva R., Yang X.O., Martinez G., Zhang Y., Panopoulos A.D., Ma L., Schluns K., Tian Q., Watowich S.S., Jetten A.M. (2007). Essential autocrine regulation by IL-21 in the generation of inflammatory T cells. Nature.

[76] Zhou L., Ivanov I., Spolski R., Min R., Shenderov K., Egawa T., Levy D.E., Leonard W.J., Littman D.R. (2007). IL-6 programs T(H)-17 cell differentiation by promoting sequential engagement of the IL-21 and IL-23 pathways. Nat. Immunol..

[77] Chung Y., Chang S.H., Martinez G.J., Yang X.O., Nurieva R., Kang H.S., Ma L., Watowich S.S., Jetten A.M., Tian Q. (2009). Critical regulation of early Th17 cell differentiation by interleukin-1 signaling. Immunity.

[78] Dong C. (2021). Cytokine Regulation and Function in T Cells. Annu. Rev. Immunol..

[79] Dong C., Flavell R.A. (2001). Th1 and Th2 cells. Curr. Opin. Hematol..

[80] Asadzadeh Z., Mohammadi H., Safarzadeh E., Hemmatzadeh M., Mahdian-Shakib A., Jadidi-Niaragh F., Azizi G., Baradaran B. (2017). The paradox of Th17 cell functions in tumor immunity. Cell. Immunol..

[81] Ivanov I.I., McKenzie B.S., Zhou L., Tadokoro C.E., Lepelley A., Lafaille J.J., Cua D.J., Littman D.R. (2006). The Orphan Nuclear Receptor RORγt Directs the Differentiation Program of Proinflammatory IL-17+ T Helper Cells. Cell.

[82] Mohammadi H., Sharafkandi N., Hemmatzadeh M., Azizi G., Karimi M., Jadidi-Niaragh F., Baradaran B., Babaloo Z. (2018). The role of innate lymphoid cells in health and disease. J. Cell. Physiol..

[83] Wang L., Yi T., Kortylewski M., Pardoll D.M., Zeng D., Yu H. (2009). IL-17 can promote tumor growth through an IL-6-Stat3 signaling pathway. J. Exp. Med..

[84] Pelletier M., Maggi L., Micheletti A., Lazzeri E., Tamassia N., Costantini C., Cosmi L., Lunardi C., Annunziato F., Romagnani S. (2010). Evidence for a cross-talk between human neutrophils and Th17 cells. Blood.

[85] Coward J., Kulbe H., Chakravarty P., Leader D., Vassileva V., Leinster D.A., Thompson R., Schioppa T., Nemeth J., Vermeulen J. (2011). Interleukin-6 as a Therapeutic Target in Human Ovarian Cancer. Clin. Cancer Res..

[86] Angevin E., Tabernero J., Elez E., Cohen S.J., Bahleda R., van Laethem J.L., Ottensmeier C., Lopez-Martin J.A., Clive S., Joly F. (2014). A phase I/II, multiple-dose, dose-escalation study of siltuximab, an anti-interleukin-6 monoclonal antibody, in patients with advanced solid tumors. Clin. Cancer Res. Off. J. Am. Assoc. Cancer Res..

[87] Gowhari Shabgah A., Amir A., Gardanova Z.R., Olegovna Zekiy A., Thangavelu L., Ebrahimi Nik M., Ahmadi M., Gholizadeh Navashenaq J. (2021). Interleukin-25: New perspective and state-of-the-art in cancer prognosis and treatment approaches. Cancer Med..

[88] Picciani B.L.S., Dziedzic A., Werneck J.T., Marinho M.A., Dick T.N.A., Quintanilha N.R., Dias E.P. (2021). Atypical oral candidiasis in a psoriatic patient during targeted immunotherapy with an interleukin 17 inhibitor (secukinumab). BMC Oral Health.

[89] Monsour E.P., Pothen J., Balaraman R. (2019). A Novel Approach to the Treatment of Pembrolizumab-induced Psoriasis Exacerbation: A Case Report. Cureus.

[90] Ridker P.M., MacFadyen J.G., Thuren T., Everett B.M., Libby P., Glynn R.J. (2017). Effect of interleukin-1β inhibition with canakinumab on incident lung cancer in patients with atherosclerosis: Exploratory results from a randomised, double-blind, placebo-controlled trial. Lancet.

[91] Wong C.C., Baum J., Silvestro A., Beste M.T., Bharani-Dharan B., Xu S., Wang Y.A., Wang X., Prescott M.F., Krajkovich L. (2020). Inhibition of IL1β by Canakinumab May Be Effective against Diverse Molecular Subtypes of Lung Cancer: An Exploratory Analysis of the CANTOS Trial. Cancer Res..

[92] Ono T., Hayashi M., Sasaki F., Nakashima T. (2020). RANKL biology: Bone metabolism, the immune system, and beyond. Inflamm. Regen..

[93] Scagliotti G.V., Hirsh V., Siena S., Henry D.H., Woll P.J., Manegold C., Solal-Celigny P., Rodriguez G., Krzakowski M., Mehta N.D. (2012). Overall survival improvement in patients with lung cancer and bone metastases treated with denosumab versus zoledronic acid: Subgroup analysis from a randomized phase 3 study. J. Thorac. Oncol. Off. Publ. Int. Assoc. Study Lung Cancer.

[94] Margolin K., Morishima C., Velcheti V., Miller J.S., Lee S.M., Silk A.W., Holtan S.G., Lacroix A.M., Fling S.P., Kaiser J.C. (2018). Phase I Trial of ALT-803, A Novel Recombinant IL15 Complex, in Patients with Advanced Solid Tumors. Clin. Cancer Res. Off. J. Am. Assoc. Cancer Res..

[95] Liu X., Nurieva R.I., Dong C. (2013). Transcriptional regulation of follicular T-helper (Tfh) cells. Immunol. Rev..

[96] Choi J.K., Egwuagu C.E. (2021). Interleukin 35 Regulatory B Cells. J. Mol. Biol..

[97] Pérol L., Lindner J.M., Caudana P., Nunez N.G., Baeyens A., Valle A., Sedlik C., Loirat D., Boyer O., Créange A. (2016). Loss of immune tolerance to IL-2 in type 1 diabetes. Nat. Commun..

[98] Bo M., Niegowska M., Erre G.L., Piras M., Longu M.G., Manchia P., Manca M., Passiu G., Sechi L.A. (2018). Rheumatoid arthritis patient antibodies highly recognize IL-2 in the immune response pathway involving IRF5 and EBV antigens. Sci. Rep..

[99] Bo M., Niegowska M., Frau J., Sechi G., Arru G., Cocco E., Sechi L.A. (2020). IL-2 and Mycobacterial Lipoarabinomannan as Targets of Immune Responses in Multiple Sclerosis Patients. Microorganisms.

[100] Abbas A.K., Trotta E., Simeonov D.R., Marson A., Bluestone J.A. (2018). Revisiting IL-2: Biology and therapeutic prospects. Sci. Immunol..

[101] Ohue Y., Nishikawa H. (2019). Regulatory T (Treg) cells in cancer: Can Treg cells be a new therapeutic target?. Cancer Sci..

[102] Silva D.-A., Yu S., Ulge U.Y., Spangler J.B., Jude K.M., Labao-Almeida C., Ali L.R., Quijano-Rubio A., Ruterbusch M., Leung I. (2019). De novo design of potent and selective mimics of IL-2 and IL-15. Nature.

[103] Seidel J.A., Otsuka A., Kabashima K. (2018). Anti-PD-1 and Anti-CTLA-4 Therapies in Cancer: Mechanisms of Action, efficacy, and Limitations. Front. Oncol..

[104] Wing K., Onishi Y., Prieto-Martin P., Yamaguchi T., Miyara M., Fehervari Z., Nomura T., Sakaguchi S. (2008). CTLA-4 control over Foxp3+ regulatory T cell function. Science.

[105] Hodi F.S., Mihm M.C., Soiffer R.J., Haluska F.G., Butler M., Seiden M.V., Davis T., Henry-Spires R., MacRae S., Willman A. (2003). Biologic activity of cytotoxic T lymphocyte-associated antigen 4 antibody blockade in previously vaccinated metastatic melanoma and ovarian carcinoma patients. Proc. Natl. Acad. Sci. USA.

[106] Herbst R.S., Giaccone G., de Marinis F., Reinmuth N., Vergnenegre A., Barrios C.H., Morise M., Felip E., Andric Z., Geater S. (2020). Atezolizumab for First-Line Treatment of PD-L1-Selected Patients with NSCLC. N. Engl. J. Med..

[107] Yang J.J., Huang C., Fan Y., Pan H., Feng J., Jiang L., Li X.Y., Liu X.Q., Xiong J.P., Zhao Y.Q. (2021). Camrelizumab in different PD-L1 expression cohorts of pre-treated advanced or metastatic non-small cell lung cancer: A phase II study. Cancer Immunol. Immunother. CII.

[108] Zhou N., Jiang M., Li T., Zhu J., Liu K., Hou H., Zhang X. (2021). Anlotinib combined with anti-PD-1 antibody, camrelizumab for advanced NSCLCs after multiple lines treatment: An open-label, dose escalation and expansion study. Lung Cancer.

[109] Antonia S.J., Villegas A., Daniel D., Vicente D., Murakami S., Hui R., Yokoi T., Chiappori A., Lee K.H., de Wit M. (2017). Durvalumab after Chemoradiotherapy in Stage III Non-Small-Cell Lung Cancer. N. Engl. J. Med..

[110] Fitzpatrick O., Naidoo J. (2021). Immunotherapy for Stage III NSCLC: Durvalumab and Beyond. Lung Cancer.

[111] Chao M.P., Takimoto C.H., Feng D.D., McKenna K., Gip P., Liu J., Volkmer J.P., Weissman I.L., Majeti R. (2019). Therapeutic Targeting of the Macrophage Immune Checkpoint CD47 in Myeloid Malignancies. Front. Oncol..

[112] Weiskopf K. (2017). Cancer immunotherapy targeting the CD47/SIRP alpha axis. Eur. J. Cancer.

[113] Kharitonenkov A., Chen Z., Sures I., Wang H., Schilling J., Ullrich A. (1997). A family of proteins that inhibit signalling through tyrosine kinase receptors. Nature.

[114] Oldenborg P.A., Zheleznyak A., Fang Y.F., Lagenaur C.F., Gresham H.D., Lindberg F.P. (2000). Role of CD47 as a marker of self on red blood cells. Science.

[115] Blazar B.R., Lindberg F.P., Ingulli E., Panoskaltsis-Mortari A., Oldenborg P.A., Iizuka K., Yokoyama W.M., Taylor P.A. (2001). CD47 (integrin-associated protein) engagement of dendritic cell and macrophage counterreceptors is required to prevent the clearance of donor lymphohematopoietic cells. J. Exp. Med..

[116] Tsai R.K., Discher D.E. (2008). Inhibition of "self" engulfment through deactivation of myosin-II at the phagocytic synapse between human cells. J. Cell Biol..

[117] Liu J., Wang L., Zhao F., Tseng S., Narayanan C., Shura L., Willingham S., Howard M., Prohaska S., Volkmer J. (2015). Pre-Clinical Development of a Humanized Anti-CD47 Antibody with Anti-Cancer Therapeutic Potential. PLoS ONE.

[118] Kansara R.R., Speziali C. (2020). Immunotherapy in hematologic malignancies. Curr. Oncol..

[119] Sorensen M.R., Holst P.J., Steffensen M.A., Christensen J.P., Thomsen A.R. (2010). Adenoviral vaccination combined with CD40 stimulation and CTLA-4 blockage can lead to complete tumor regression in a murine melanoma model. Vaccine.

[120] Schraven B., Kalinke U. (2008). CD28 superagonists: What makes the difference in humans?. Immunity.

[121] Ramjiawan R.R., Griffioen A.W., Duda D.G. (2017). Anti-angiogenesis for cancer revisited: Is there a role for combinations with immunotherapy?. Angiogenesis.

[122] Ferrara N. (2016). VEGF and Intraocular Neovascularization: From Discovery to Therapy. Transl. Vis. Sci. Technol..

[123] Apte R.S., Chen D.S., Ferrara N. (2019). VEGF in Signaling and Disease: Beyond Discovery and Development. Cell.

[124] Presta L.G., Chen H., O’onnor S.J., Chisholm V., Meng Y.G., Krummen L., Winkler M., Ferrara N. (1997). Humanization of an anti-vascular endothelial growth factor monoclonal antibody for the therapy of solid tumors and other disorders. Cancer Res..

[125] Schlaeppi J.-M., Wood J.M. (1999). Targeting Vascular Endothelial Growth Factor (VEGF) for Anti-tumor Therapy, by Anti-VEGF Neutralizing Monoclonal Antibodies or by VEGF Receptor Tyrosine-kinase Inhibitors. Cancer Metastasis Rev..

[126] Ferrara N., Adamis A.P. (2016). Ten years of anti-vascular endothelial growth factor therapy. Nat. Rev. Drug Discov..

[127] Rama N., Dubrac A., Mathivet T., RA N.C., Genet G., Cristofaro B., Pibouin-Fragner L., Ma L., Eichmann A., Chédotal A. (2015). Slit2 signaling through Robo1 and Robo2 is required for retinal neovascularization. Nat. Med..

[128] Zhang L., Qin Y., Wu G., Wang J., Cao J., Wang Y., Wu D., Yang K., Zhao Z., He L. (2020). PRRG4 promotes breast cancer metastasis through the recruitment of NEDD4 and downregulation of Robo1. Oncogene.

[129] Li Q., Cao J., He Y., Liu X., Mao G., Wei B., Liao S., Zhang Q., Li J., Zheng L. (2020). R5, a neutralizing antibody to Robo1, suppresses breast cancer growth and metastasis by inhibiting angiogenesis via down-regulating filamin A. Exp. Cell Res..

[130] Koyanagi T., Suzuki Y., Komori K., Saga Y., Matsubara S., Fujiwara H., Sato Y. (2017). Targeting human vasohibin-2 by a neutralizing monoclonal antibody for anti-cancer treatment. Cancer Sci..

[131] Bergers G., Hanahan D. (2008). Modes of resistance to anti-angiogenic therapy. Nat. Rev. Cancer.

[132] Visvader J.E., Lindeman G.J. (2008). Cancer stem cells in solid tumours: Accumulating evidence and unresolved questions. Nat. Rev. Cancer.

[133] Kurtova A.V., Xiao J., Mo Q., Pazhanisamy S., Krasnow R., Lerner S.P., Chen F., Roh T.T., Lay E., Ho P.L. (2015). Blocking PGE(2)-induced tumour repopulation abrogates bladder cancer chemoresistance. Nature.

[134] Zhang Q., Xiao M., Gu S., Xu Y., Liu T., Li H., Yu Y., Qin L., Zhu Y., Chen F. (2019). ALK phosphorylates SMAD4 on tyrosine to disable TGF-β tumour suppressor functions. Nat. Cell Biol..

[135] Ciardiello F., Tortora G. (2008). EGFR antagonists in cancer treatment. N. Engl. J. Med..

[136] Smith H.A., Kang Y. (2013). The metastasis-promoting roles of tumor-associated immune cells. J. Mol. Med..

[137] Kho V.M., Mekers V.E., Span P.N., Bussink J., Adema G.J. (2021). Radiotherapy and cGAS/STING signaling: Impact on MDSCs in the tumor microenvironment. Cell. Immunol..

[138] Vanpouille-Box C., Formenti S.C., Demaria S. (2017). TREX1 dictates the immune fate of irradiated cancer cells. Oncoimmunology.

[139] Sterne J.A.C., Murthy S., Diaz J.V., Slutsky A.S., Villar J., Angus D.C., Annane D., Azevedo L.C.P., Berwanger O., Cavalcanti A.B. (2020). Association Between Administration of Systemic Corticosteroids and Mortality Among Critically Ill Patients With COVID-19: A Meta-analysis. JAMA.

[140] Mohamed A.J., Yu L., Bäckesjö C.M., Vargas L., Faryal R., Aints A., Christensson B., Berglöf A., Vihinen M., Nore B.F. (2009). Bruton’s tyrosine kinase (Btk): Function, regulation, and transformation with special emphasis on the PH domain. Immunol. Rev..

[141] Qiu J., Fu Y., Chen Z., Zhang L., Li L., Liang D., Wei F., Wen Z., Wang Y., Liang S. (2021). BTK Promotes Atherosclerosis by Regulating Oxidative Stress, Mitochondrial Injury, and ER Stress of Macrophages. Oxidative Med. Cell. Longev..

[142] Stack M., Sacco K., Castagnoli R., Livinski A.A., Notarangelo L.D., Lionakis M.S. (2021). BTK inhibitors for Severe Acute Respiratory Syndrome Coronavirus 2 (SARS-CoV-2): A Systematic Review. Res. Sq..

[143] Bestle D., Heindl M.R., Limburg H., Van Lam V.T., Pilgram O., Moulton H., Stein D.A., Hardes K., Eickmann M., Dolnik O. (2020). TMPRSS2 and furin are both essential for proteolytic activation of SARS-CoV-2 in human airway cells. Life Sci. Alliance.

[144] Tomlins S.A., Day J.R., Lonigro R.J., Hovelson D.H., Siddiqui J., Kunju L.P., Dunn R.L., Meyer S., Hodge P., Groskopf J. (2016). Urine TMPRSS2:ERG Plus PCA3 for Individualized Prostate Cancer Risk Assessment. Eur. Urol..

[145] Ko C.J., Huang C.C., Lin H.Y., Juan C.P., Lan S.W., Shyu H.Y., Wu S.R., Hsiao P.W., Huang H.P., Shun C.T. (2015). Androgen-Induced TMPRSS2 Activates Matriptase and Promotes Extracellular Matrix Degradation, Prostate Cancer Cell Invasion, Tumor Growth, and Metastasis. Cancer Res..

[146] Kobe H., Tachikawa R., Masuno Y., Matsunashi A., Murata S., Hagimoto H., Tomii K. (2021). Apalutamide-induced severe interstitial lung disease: A report of two cases from Japan. Respir. Investig..

[147] Tang Z., Kang B., Li C., Chen T., Zhang Z. (2019). GEPIA2: An enhanced web server for large-scale expression profiling and interactive analysis. Nucleic Acids Res..

[148] Kong Q., Xiang Z., Wu Y., Gu Y., Guo J., Geng F. (2020). Analysis of the susceptibility of lung cancer patients to SARS-CoV-2 infection. Mol. Cancer.

[149] Radzikowska U., Ding M., Tan G., Zhakparov D., Peng Y., Wawrzyniak P., Wang M., Li S., Morita H., Altunbulakli C. (2020). Distribution of ACE2, CD147, CD26, and other SARS-CoV-2 associated molecules in tissues and immune cells in health and in asthma, COPD, obesity, hypertension, and COVID-19 risk factors. Allergy.

[150] Baruah V., Bose S. (2020). Immunoinformatics-aided identification of T cell and B cell epitopes in the surface glycoprotein of 2019-nCoV. J. Med. Virol..

[151] Gretebeck L.M., Subbarao K. (2015). Animal models for SARS and MERS coronaviruses. Curr. Opin. Virol..

[152] Planas D., Veyer D., Baidaliuk A., Staropoli I., Guivel-Benhassine F., Rajah M.M., Planchais C., Porrot F., Robillard N., Puech J. (2021). Reduced sensitivity of SARS-CoV-2 variant Delta to antibody neutralization. Nature.

[153] Van Kerkhove M.D., Ryan M.J., Ghebreyesus T.A. (2021). Preparing for “Disease X”. Science.

